# Supplementary material for: Can machine learning methods be used for identification of at-risk neonates in low-resource settings? A prospective cohort study
Source: BMJ Paediatr Open. 2023 Nov 2;7(1):e002134. doi: 10.1136/bmjpo-2023-002134 (PMC10626794; doi:10.1136/bmjpo-2023-002134)

SUPPLEMENTARY MATERIAL

Figure S1: CHD screening table using pulse oximetry value on right hand and either foot

Right Hand	Either Foot											
100	100	99	98	97	96	95	94	93	92	91	90	<90
99	100	99	98	97	96	95	94	93	92	91	90	<90
98	100	99	98	97	96	95	94	93	92	91	90	<90
97	100	99	98	97	96	95	94	93	92	91	90	<90
96	100	99	98	97	96	95	94	93	92	91	90	<90
95	100	99	98	97	96	95	94	93	92	91	90	<90
94	100	99	98	97	96	95	94	93	92	91	90	<90
93	100	99	98	97	96	95	94	93	92	91	90	<90
92	100	99	98	97	96	95	94	93	92	91	90	<90
91	100	99	98	97	96	95	94	93	92	91	90	<90
90	100	99	98	97	96	95	94	93	92	91	90	<90
<90	100	99	98	97	96	95	94	93	92	91	90	<90

Table S1: For AllML, the following clinical variables were used:

Group	Variable
Maternal characteristics	Age
	Years of education
	Smoking
	Sniffing/chewing of tobacco
	Chewing of betel nut
	Cardiac disease
	Diabetes
	Thyroid disease
	Any type of cancer
	Epilepsy
Obstetric History	Number of pregnancies
	Number of pregnancies ended before 7 months
	Number of stillborn
	Number of deliveries
	Number of live deliveries
	Number of children died during delivery
	Number of living children
	Number of children died
	Number of children with congenital heart disease
	Number of children with any heart surgery
Current pregnancy characteristics	Received antenatal care from a skilled care
	Vaginal bleeding
	High blood pressure
	Convulsions
	Diabetes
	Anemia
	Fever
	Blood transfusion
	Overnight admission to a health facility
Neonatal characteristics	Gestational age
	Delayed cry
	Weight
	Gender
	Place of birth
	Mode of delivery

Table S2: Details of the study participants who died at four weeks of age

S.No	Mode of delivery	Place of delivery	GA at time of delivery (weeks)	Birth weight (kg)	Age at time of enrollment (hours)	WHO Signs	Pulse Oxi-meter	ECHO done	Age at death (in days)	Place of death	Cause of death
**1**	SVD	Clinic	32	2.53	43	Pass	Pass	No	8	Hospital	Neonatal sepsis
**2**	SVD	Hospital	40	2.59	36	Pass	Pass	No	19	Home	Neonatal sepsis
**3**	SVD	Home	40	1.92	6	Fail	Fail	Yes*	13	Home	Neonatal sepsis
**4**	SVD	Hospital	25	1.19	49	Pass	Pass	No	16	Home	Pre-term related complicatio n
**5**	SVD	Clinic	38	2.00	11	Fail	Pass	No (refused)	6	Hospital	Neonatal sepsis
**6**	SVD	Hospital	35	1.92	34	Pass	Pass	No	3	Home	Not determined after VA
**7**	SVD	Hospital	40	2.86	69	Fail	Pass	No (refused)	3	Hospital	Neonatal sepsis
**8**	SVD	Clinic	38	2.96	58	Pass	Pass	No	6	Hospital	Neonatal tetanus
**9**	SVD	Hospital	33	2.34	78	Pass	Pass	No	30	Home	Not determined after VA
**10**	SVD	Clinic	40	4.21	21	Pass	Pass	No	5	Home	Not determined after VA
**11**	SVD	Home	34	1.86	37	Pass	Pass	No	9	Home	Not determined after VA
**12**	SVD	Home	39	2.76	11	Pass	Pass	No	11	Home	Neonatal sepsis
**13**	SVD	Home	51	2.08	31	Pass	Pass	No	28	Hospital	Neonatal sepsis

**14**	Cesarean section	Hospital	36	3.29	96	Pass	Pass	No	20	Home	Not determined after VA
**15**	SVD	Home	40	2.73	18	Pass	Pass	No	26	Home	Neonatal sepsis
**16**	SVD	Hospital	39	2.56	67	Pass	Fail	Yes^	21	Home	Neonatal pneumonia
**17**	Cesarean section	Hospital	36	2.56	94	Pass	Pass	No	18	Home	Neonatal sepsis

*Severe tricuspid regurgitation, ^patent foramen ovale – both ECHOs were performed on the same day of assessment; VA – verbal autopsy;

Table S3: Sensitivity and specificity of the pulse oximeter in detecting CCHD or neonatal sepsis

	**For CCHD detection (n=1317)**	**For detection of neonatal sepsis (n=1317)**
True positives (n)	0	9
True negatives (n)	1289	1259
False positives (n)	26	17
False negatives (n)	2	32
Sensitivity (95% CI) (%)	0 (0-84.2)	21.9 (10.6-37.6)
Specificity (95% CI) (%)	98 (97.1-98.7)	98.6 (97.9-99.2)

The two cases of false negative CCHD were Tetralogy of Fallot and isolated valvar pulmonary stenosis. In both these children, the pulse oximeter readings were above 95% were hand and foot screening.

Table S4: Performance metrics of the different machine learning models developed on the clinical, WHO signs and pulse oximetry data

	**POx**		**WHO**		**POx | WHO**		**CliML**		**SeqML**		**AllML**	
	**Train**	**Test**	**Train**	**Test**	**Train**	**Test**	**Train**	**Test**	**Train**	**Test**	**Train**	**Test**
**TP**	7	5	27	17	29	19	37	12	40	23	37	17
**FP**	10	4	5	5	14	9	153	118	165	124	21	15
**FN**	34	22	14	10	12	8	4	15	1	4	4	10
**TN**	739	496	744	495	735	491	596	382	584	376	728	485
**SE**	17	19	66	63	71	70	90	44	98	85	90	63
**SP**	99	99	99	99	98	98	80	76	78	75	97	97
**AUC**	0.58	0.59	0.83	0.81	0.84	0.84	0.85	0.60	0.88	0.80	0.94	0.80
**F1**	0.24	0.28	0.74	0.69	0.69	0.69	0.32	0.15	0.33	0.26	0.75	0.58
**PR**	0.41	0.56	0.84	0.77	0.67	0.68	0.19	0.09	0.20	0.16	0.64	0.53

POx: pulse-oximetry alone; WHO: WHO signs alone; POx | WHO: POx or WHO signs; CliML: ML model for clinical data only; SeqML: sequential use of POx, WHO and CliML; AllML: ML model for the clinical data enriched with WHI signs outcome and POx values (saturations of hand and toe and heart rate); Train: Results for the training set; Test: results for the test set; TP: True Positives; FP: False Positives; FN: False Negatives; TN: True Negatives; SE: Sensitivity; SP: Specificity; AUC: Area under the Curve; F1: F1 score; PR: Precision)

Figure S2: SHapley Additive exPlanations (SHAP) analysis for the ML model based on the clinical variables, showing the main feature used in the ML model that contribute most to the decision. The higher the SHAP value, the more important the feature. A positive value contributes to a positive decision (at-risk neonate), while a more negative value suggest the opposite. A red color indicates a high value for the feature, while a blue color corresponds to a low value. For example, from the top features we can see that a low weight and a low gestational age is associated to a more at-risk neonate.

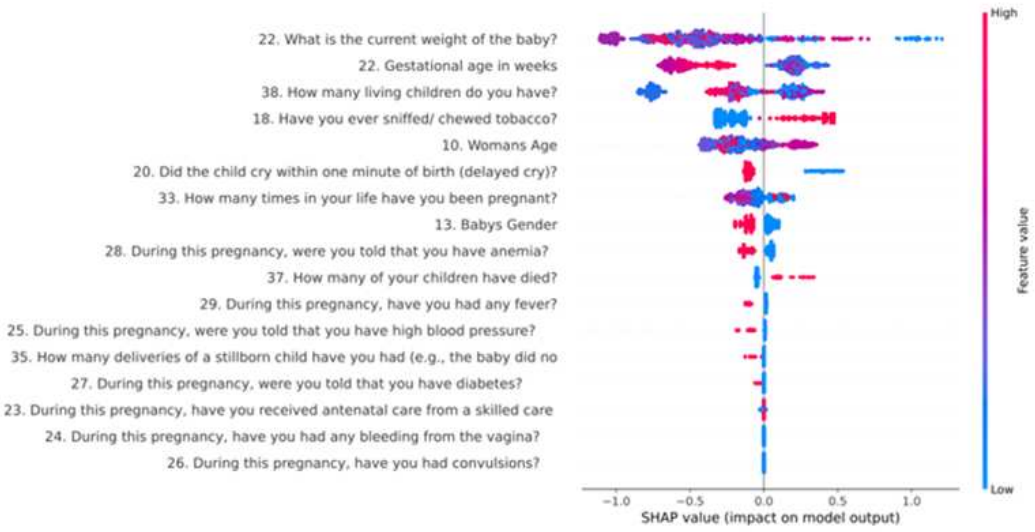

Figure S3: ROC analysis for the ML based on clinical variables as well as the sequential use of WHOS, POx and clinical ML

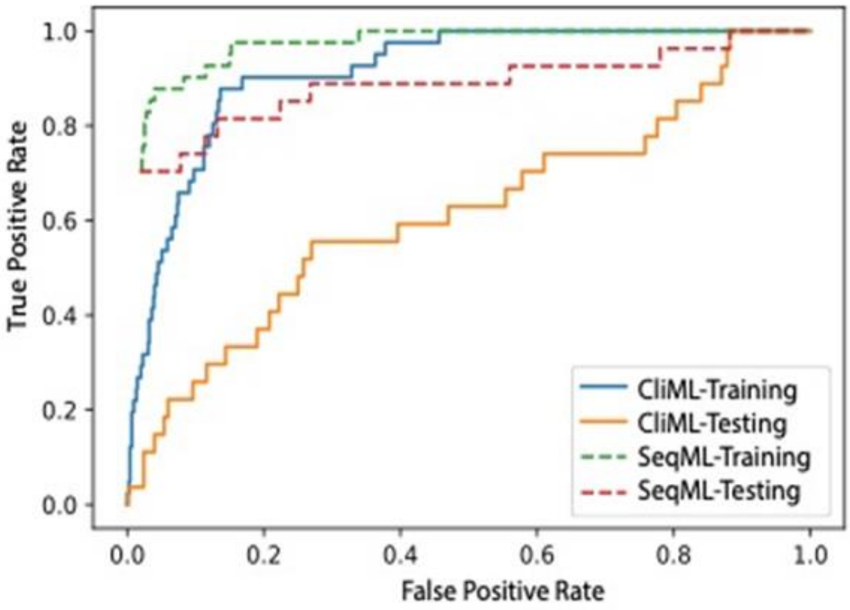

Supplement: Supplementary data [file bmjpo-2023-002134supp001.pdf]
