# Supplementary material for: Can machine learning methods be used for identification of at-risk neonates in low-resource settings? A prospective cohort study
Source: BMJ Paediatr Open. 2023 Nov 2;7(1):e002134. doi: 10.1136/bmjpo-2023-002134 (PMC10626794; doi:10.1136/bmjpo-2023-002134)

BMJ Paediatrics Open

BMJ Paediatrics Open is committed to open peer review. As part of this commitment we make the peer review history of every article we publish publicly available.

When an article is published we post the peer reviewers' comments and the authors' responses online. We also post the versions of the paper that were used during peer review. These are the versions that the peer review comments apply to.

The versions of the paper that follow are the versions that were submitted during the peer review process. They are not the versions of record or the final published versions. They should not be cited or distributed as the published version of this manuscript.

BMJ Paediatrics Open is an open access journal and the full, final, typeset and author-corrected version of record of the manuscript is available on our site with no access controls, subscription charges or pay-per-view fees (http://bmjpaedsopen.bmj.com).

If you have any questions on BMJ Paediatrics Open's open peer review process please email info.bmjpo@bmj.com

BMJ Paediatrics Open

A sequential approach of classical screening enriched with machine learning improves neonatal wellness screening in low resource settings: a prospective cohort study

Journal:	*BMJ Paediatrics Open*
Manuscript ID	bmjpo-2023-002134
Article Type:	Original research
Date Submitted by the Author:	13-Jun-2023
Complete List of Authors:	Hasan, Babar; Sindh Institute of Urology and Transplantation, Department of Pediatrics and Child Health<br>Hoodbhoy, Zahra; Aga Khan University, Department of Pediatrics and Child Health<br>Khan, Amna; The Aga Khan University, Department of Pediatrics and Child Health<br>Nogueira, Mariana; IDIBAPS<br>Bijnens, Bart; IDIBAPS; ICREA<br>Chowdhury, Devyani; The Children's Hospital of Philadelphia
Keywords:	Screening, Neonatology

SCHOLARONE™
Manuscripts

I, the Submitting Author has the right to grant and does grant on behalf of all authors of the Work (as defined in the below author licence), an exclusive licence and/or a non-exclusive licence for contributions from authors who are: i) UK Crown employees; ii) where BMJ has agreed a CC-BY licence shall apply, and/or iii) in accordance with the terms applicable for US Federal Government officers or employees acting as part of their official duties; on a worldwide, perpetual, irrevocable, royalty-free basis to BMJ Publishing Group Ltd ("BMJ") its licensees and where the relevant Journal is co-owned by BMJ to the co-owners of the Journal, to publish the Work in this journal and any other BMJ products and to exploit all rights, as set out in our licence.

The Submitting Author accepts and understands that any supply made under these terms is made by BMJ to the Submitting Author unless you are acting as an employee on behalf of your employer or a postgraduate student of an affiliated institution which is paying any applicable article publishing charge ("APC") for Open Access articles. Where the Submitting Author wishes to make the Work available on an Open Access basis (and intends to pay the relevant APC), the terms of reuse of such Open Access shall be governed by a Creative Commons licence – details of these licences and which Creative Commons licence will apply to this Work are set out in our licence referred to above.

Other than as permitted in any relevant BMJ Author's Self Archiving Policies, I confirm this Work has not been accepted for publication elsewhere, is not being considered for publication elsewhere and does not duplicate material already published. I confirm all authors consent to publication of this Work and authorise the granting of this licence.

Confidential: For Review Only

Title: A sequential approach of classical screening enriched with machine learning improves neonatal wellness screening in low resource settings: a prospective cohort study

Authors:

Babar Sultan Hasan*[1] MBBS, Zahra Hoodbhoy*[2] PhD, Amna Khan[2] BA, Mariana Nogueira[3] PhD, Bart Bijnens PhD[3,4], Devyani Chowdhury[5,6] MD

*Shared first authorship

#Shared senior authorship

[1]Department of Pediatrics and Child Health, Stadium Road, The Aga Khan University, Karachi, Pakistan relocated to Sindh Institute of Urology and Transplantation (SIUT), Karachi, Pakistan

[2]Department of Pediatrics and Child Health, Stadium Road, The Aga Khan University, Karachi, Pakistan

[3]IDIBAPS (August Pi i Sunyer Biomedical Research Institute), Barcelona, Spain

[4]ICREA, Barcelona, Spain

[5]Cardiology Care for Children, Pennsylvania, USA,

[6]Nemours Cardiac Center, Wilmington, DE

Corresponding author:

Babar Hasan
Professor and Chair
Division of Cardiothoracic Sciences
Sindh Institute of Urology and Transplantation (SIUT)
Karachi, Pakistan
Tel No: +92-3362559473
Email address: drbabarhasan@yahoo.com

Confidential: For Review Only

Abstract:

Introduction: Timely identification of at-risk neonates (ARN) in the community is essential to reduce mortality in low resource settings. Tools such as AAP pulse-oximetry (POx) and WHO Young Infants Clinical Signs (WHOS) have high specificity but low sensitivity to identify at risk neonates (ARN). Our aim was assessing the value of POx and WHOS independently, in combination, and with machine learning (ML) from clinical features, to detect ARN in a low- to middle-income country (LMIC)

Methods: This prospective cohort study was conducted in a peri-urban community in Pakistan. Eligible live births were screened using WHOS and POx along with clinical information regarding pregnancy and delivery. The enrolled neonates were followed for 4 weeks of life to assess the vital status. The predictive value to identify ARN, of POx, WHOS and an ML model using maternal and neonatal clinical features was assessed.

Results: Of 1336 neonates, 68 (5%) had adverse outcomes, i.e. sepsis (n=40, 59%), critical congenital heart disease (n=2, 3%), severe persistent pulmonary hypertension (n=1), hospitalization (n=8, 12%) and death (n=17, 25%). Specificity (SP) of POx and WHOS to independently identify ARN was 99%, with sensitivity (SE) of 19% and 63% respectively. Combining both improved SE to 70%, keeping SP at 98%. An ML model using clinical variables had 44% SE and 76% SP. A staged assessment, where WHOs, POx and ML are sequentially used for triage increased SE to 85%, keeping SP 75%. Using ML (when WHO and POx negative) for community follow-up detected the majority of ARN.

Conclusion: Classical screening combined with ML, can help maximizing identifying ARN and could be embedded in low-resource clinical settings, thereby improving outcome. Sequential use

Confidential: For Review Only

of classical assessment and clinical ML identifies the most at-risk neonates in the community, still optimizing follow-up clinical care.

Keywords: neonatal wellness; screening; community health workers; machine learning

Confidential: For Review Only

Introduction:

With a neonatal mortality rate (NMR) of 42 per 1000 live births, Pakistan accounts for 7% of all global neonatal deaths and ranks 3rd on the list among countries with highest NMR[1]. The top four causes of neonatal deaths are pre-maturity, birth asphyxia, sepsis and congenital anomalies, most of which are congenital heart diseases (CHD)[2].

Newborn screening at few hours of life, for identification of at-risk newborns (ARN), has been proposed as an effective tool to reduce NMR[3]. In high-income countries (HICs), a combination of antenatal ultrasound and newborn screening enables early detection, diagnosis, and intervention to prevent death or disability. Newborn screening, using a few drops of blood for genetic, metabolic and endocrine disorders, and American Academy of Pediatrics (AAP) pulse oximetry (POx) for critical congenital heart defects (CCHDs) are widely implemented in HICs[4].

While POx is a highly specific tool (specificity = 99.9%) to detect CCHD[5], it will detect other conditions associated with hypoxia, such as neonatal sepsis, hypothermia and persistent pulmonary hypertension[6], where it has demonstrated a high specificity (>99.9%) but low sensitivity (42%)[7, 8]. POx thus has shown a broader utility as a newborn wellness screen, with a good feasibility and acceptability for screening newborns in primary care centers in low middle income countries (LMICs)[9].

A complementary newborn wellness screening tool widely used in LMICs, is the WHO Young Infants Clinical Signs Study Group (WHOS), which identifies seven signs including fever ≥ 38°C, hypothermia (temperature < 35°C), convulsions, lethargy, poor feeding, chest indrawing and tachypnea (respiratory rate > 60 breaths per minute) and has a sensitivity of 74% and specificity of 79% to predict severe illness[10]. Though WHOS and POx have shown only modest

Confidential: For Review Only

sensitivity, these are simple and highly specific screening tools which may be used to identify newborn conditions contributing to NMR in low resource settings.

Given their moderate sensitivity, there remains a need to optimize the performance of these tools and to develop improved strategies by including the use of a wider set of clinical parameters for community-based triage of ARN[10]. The aim of this study was to assess the value of POx and WHOS, used on their own, in combination, and enriched with machine learning (ML) from clinical features, to develop a screening tool for community health workers (CHWs) to identify ARN.

Confidential: For Review Only

Methods:

Study design and participants:

This was an ethics committee approved (Aga Khan University ERC number: 2019-0551-4976) single group prospective cohort study conducted at Ibrahim Hyderi (IH), a peri-urban settlement of approximately 100,000 individuals on the south-east of Karachi, Pakistan, from October 1, 2020 to March 31, 2022. The main occupation of individuals in this area is fishing with 40% below the poverty line[11]. The NMR at this site ranges between 40-45 per 1000 live births[11]. The eligibility criterion was a single live birth where the mother planned to stay in the study catchment area for at least four weeks after delivery.

Sample size estimation:

The sample size was estimated based on the primary outcomes of this study. Neonatal sepsis has been reported to be as high as 14%[2] while the prevalence of CHD in Pakistan is stated to be around 0.9%[12]. With a 95% confidence interval, power of 80% and absolute precision of 0.6%, a frequency of 0.9% generated the maximum sample size of 951 neonates. To account for 5% loss to follow up, the sample size was inflated to 1000 neonates.

Procedures:

The Department of Pediatrics and Child Health at The Aga Khan University (AKU) maintains a demographic and health surveillance system at this site whereby all households are enumerated. Leveraging the surveillance data for identification of pregnant women, trained CHWs visited these households to screen eligible women and obtained written informed consent for the participation of their newborn in the research study. All live births registered within 72 hours, where clinical information about the pregnancy was collected (including maternal biometry and co-morbidities; obstetric history, including past pregnancies; pregnancy complications such as

Confidential: For Review Only

preeclampsia, diabetes, anemia, medication use during pregnancy; delivery information, such as gestational age at delivery, place and mode of delivery, maternal and neonatal complications), were assessed for danger signs using WHOS[10] along with POx (using the Masimo Rad-8 pulse oximeter).

The oxygen saturation (SpO2) was measured on the right hand and foot (30 seconds each) once the device's signal quality index showed stable green bars for >10 seconds. The SpO2 readings (saturations and heart rate) were recorded and categorized into green, yellow and red zone as per AAP recommendations[13] (Figure S1). The algorithm shown in Figure 1 was followed. Neonates who had a positive WHOS or fell in the yellow or red zone for POx, were referred for further evaluation and echocardiography with follow up by a physician at the primary health care center (PHC). As per PHC protocol, all clinically sick neonates who were prescribed antibiotics were followed daily for the next 7 days. As part of the study, all neonates were also followed up at four weeks of life to assess the vital status of the child, and hospitalizations, if any, during this period. For all neonatal deaths, a verbal autopsy was performed using the WHO 2016 verbal autopsy instrument by trained research staff[14] and coded by a physician to ascertain cause of death.

Outcomes:

The primary outcome of this study was the identification of ARN, defined as:

- Early neonatal sepsis – Confirmed by a physician after referral based on WHOS or other clinical indications[10].
- Critical CHD (CCHDs) – Confirmed by echocardiography (transposition of the great arteries, double outlet right ventricle, complex single ventricle physiology, Tetralogy of Fallot, truncus arterious[15].

Confidential: For Review Only

- Other cardiac conditions, such as severe persistent pulmonary hypertension (PPHN) (bi-directional patent ductus arteriosus (PDA) and right ventricular dysfunction.)
- Hospitalization/death within the first four weeks of life.

Neonates with isolated non-hemodynamically significant CHDs, like small ventricular septal defects (VSD) and atrial septal defects (ASD)[16], were not considered as ARN.

Predicting ARN

To predict ARN from the data collected from WHOS, POx and the clinical parameters several models were assessed. Details on the combinations of variables used in different models are mentioned below.

Statistical analysis and Machine Learning:

Conventional data analysis was performed using STATA 16.4 bit. Data was reported as percentages and means +/- standard deviation where appropriate.

For the ML, we used the XGBoost implementation of gradient boosted decision trees[17]. For the first model (AllML), an extensive set of clinical variables (table S1), the measurements obtained from POx, and the outcome of WHOS (pass/fail) was used; for the second model (CliML), POx and WHOS were excluded with a focus on a smaller set of clinical variables selected based on a combination of prior expert knowledge on relevant features for the prediction task at hand and the results of a model explainer, ranking variables according to importance for outcome prediction in AllML. We also used the SHapley Additive exPlanations (SHAP)[18] approach to understand the outputs of the ML models. The models were trained on 60% of the data and using 3-fold cross validation. The remaining 40% were used to test how well they generalized to unseen cases. During the training stage, a grid search was performed to tune model hyperparameters, and those yielding the best cross-validation F1-score were selected. The F1-score and other

Confidential: For Review Only

performance metrics (sensitivity, specificity, precision, the Receiver Operating Characteristic (ROC) and respective Area Under the Curve (AUC) were then computed for the full training and the testing set.

Finally, a sequential use (SeqML) of WHOS, POx, and CliML was assessed to see if it could provide the basis of an alternative clinical workflow optimizing the detection of ARN. For SeqML, WHOS negative individuals undergo POx, and those passing POx would be assessed with CliML. A ROC curve was constructed using a moving threshold on the probability provided by CliML and adding this to the (fixed) sensitivities and specificities of POx after WHOS.

Confidential: For Review Only

Results:

During the study period, 1336 neonates were enrolled from the community (figure 2). Maternal and neonatal characteristics of the study population are described in Table 1. The mean maternal age was 27+/- 5.1 years, 46% (n=618) did not have any formal education, 92%(n=933) had received at least one antenatal visit during the current pregnancy and, one third (n=397) delivered at home.

Table 1: Baseline characteristics of mothers and neonates enrolled in the study

**Maternal characteristics (N=1336)**	
Maternal age (in years), mean ± SD	27+/-5.1
Literacy rate, *n (%)*	618 (46)
Unemployed, *n (%)*	1275 (95)
Number of pregnancies, Median IQR	3 (1-14)
Number of alive children, Median IQR	2 (0-10)
Received antenatal care in the current pregnancy, *n (%)*	1248 (93)
**Neonatal characteristics (N=1336)**	
GA at delivery (in weeks), mean ± SD	39+/-4.5
Preterm	313 (23)
Term	1023 (77)
Place of birth, *n (%)*	
Facility	939 (70)
Home	397 (30)
Mode of delivery, *n (%)*	
Spontaneous vaginal delivery	1015 (76)
Assisted vaginal delivery	3 (<1)
Cesarean section	318 (24)
Gender, *n (%)*	
Female	646 (48)
Male	690 (52)

Confidential: For Review Only

Hours at time of enrollment, Median IQR<br>< 24 hours<br>24-48 hours<br>48 -72 hours<br>>72 hours	48.2 (0-141)<br>256 (19)<br>462 (34.5)<br>389 (29.1)<br>229 (17.1)
Weight at enrollment in kg, Median IQR	2.84 (1.19-5.72)
Difference in hours between pulse oximeter screening and echocardiography<br>< 24 hours<br>24-72 hours<br>>72 hours	 <br>42 (60)<br>14 (20)<br>14 (20)

For the final outcome at four weeks follow up, neonates were labeled as *well* or *ARN*. There were 19 neonates who were excluded from the analysis due to refusal for follow up. We identified 68/1317 (5.1%) ARN. These included 40 cases (59%) of neonatal sepsis, 2 cases (3%) of CCHD (both Tetralogies of Fallot, one also with sepsis), 1 case (1.5%) of severe PPHN (bi-directional PDA with right ventricular dysfunction), 8 (11.5%) hospitalizations and 17 deaths (25%). The detailed characteristics of the study participants who died are described in Table S2. Of the neonatal deaths (n=17), 76% (n=13) passed both WHOS and pulse oximeter screening. Five of these (30%) had the screening performed at less than 24 hours of life. Five were early neonatal deaths with approximately 2.5 days between enrollment and death while for the 12 late neonatal deaths, the median difference between enrollment and death was 16.5 days (IQR 6-26.5).

The distribution of the neonates according to WHOS and POx screening is shown in figure 3 with more details in table S3.

ARN screening performance

1317 neonates were included in the predictive modeling to assess ARN. The training set (60%) consisted of 790 neonates while the test set included 527. Table S4 summarizes all results.

Confidential: For Review Only

As expected, WHOS (absent/present) and POx (pass/fail) showed a high specificity, but only moderate sensitivity to detect ARN. Combining both improved overall sensitivity as compared to the singular tests, while preserving specificity. When comparing the results in training and test set, they showed similar performance, confirming their stable performance in this population.

The ML model based on the clinical variables (CliML) showed good sensitivity/specificity on training, but much worse performance during testing, indicating overfitting.

AllML, trained on all assessed information at once, showed excellent performance at training, but reduced sensitivity in the test set indicating overfitting, so that it does not outperform the combination of WHOS and POx. The variables that contributed most to the decision included maternal features such as tobacco use, previous pregnancies, child deaths and pregnancy complication, as well as neonatal features, such as gender, gestational age at delivery and birthweight as indicated by the SHAP analysis (figure S2).

To find a balance between the high specificity of WHOS and POx and the potentially higher sensitivity of CliML, a sequential testing approach, composed of first assessing WHOS, followed by POx and CliML to further identify ARN was investigated (figure 4). Despite some overfitting during training, SeqML clearly showed increased sensitivity in the test set, as compared to all other approaches. Figure S3 shows the ROC curve for both CliML as well as the sequential approach of SeqML.

Confidential: For Review Only

Discussion:

High NMR in LMIC is an important public health problem that can be addressed by timely detection of ARN. There are several barriers that have been cited for poor uptake of neonatal screening in LMIC, including issues such as medical, technical and logistic support as well as planning for policy uptake[19]. Hence it is important to identify an implementation framework for such programs which includes midwives and front-line CHW who are equipped with these tools to detect ARN. These neonates can then be appropriately followed by confirmatory testing and affordable management pathways[19].

This study showed that a sequentially deployed strategy for newborn screening, to detect ARN, based on WHOS, POx, and an ML model using clinical information, can outperform the traditional approach of using only POx or WHO signs. The model identifies a maximal number of ARN and can be implemented in a staged manner where those failing WHOS and POx can be referred sooner to a higher-level care facility, while intermediate risk neonates detected by the SeqML are monitored at home by a CHW. In LMICs, this SeqML can be an important tool to reduce the unacceptably high NMR and to achieve similar results as in HIC.

WHOS in LMIC, is shown to be specific for sepsis, but has modest sensitivity to detect non-infectious etiologies. In the framework presented by the Global Initiative for Children's cardiac Surgery, PO and WHOS are a part of basic infrastructure for timely outpatient screening for cardiac disease[20]. POx is recommended for screening for CCHD and is also able to detect other hypoxia causing non-cardiac conditions[13, 21]. This has led to proposed recommendations of the WHO Integrated Management of Childhood Illnesses (IMCI) guidelines for detecting severe pneumonia to include oxygen saturation of <93% as a point for referral[22]. In this study, POx is highly specific for identifying ARN, but has a poor sensitivity when used stand-alone. Amongst

Confidential: For Review Only

factors that might contribute to this is the timing of the screening as while the ductus arteriosus is still open there is a higher sensitivity to detect life-threatening CCHD[23, 24]. In the current study, there were four cases who were screened > 24 hours after birth, did not have WHOS and passed the POx screening, but died within 7-14 days of birth at home where cause of death could not be ascertained. It may be possible that an underlying cardiac condition, especially left sided lesions, may have been missed due to late neonatal screening. Additionally, the POx screening devices used only provided readings of the saturation values and heart rate and did not have visual feedback on acquired waveforms. While out of the scope of this manuscript, when examining the stored waveforms, it was observed that the quality of tracing might have also contributed to the lower sensitivity.

Besides the outcome of WHOS and POx for screening, the study showed that their combination allows to increase sensitivity while maintaining high specificity, indicating detecting other etiologies associated with ARN. However, the modest sensitivity of the combination of both indicates that not all ARN are identified. The complementary value of easily obtainable clinical information (from the mother, her pregnancy and the neonate), was explored in CliML to aid in predicting ARN. CliML provided predictive information, but only at modest sensitivity and specificity. Training a singular ML model (AllML) using all available information at once showed potential but had problems with generalizability given that the performance in the test data was inferior to the training. The sequential screening model (SeqML) where WHOS, a specific test first identifies neonates at risk of sepsis and then identifies etiologies associated with decreased saturations, through POx, and finally identifies those at higher risks using an ML algorithm based on clinical information, was most successful in identifying ARN. This approach optimizes a staged follow-up given that WHOS and POx identify those that clearly need urgent medical attention,

Confidential: For Review Only

while the clinical data identifies an increased risk without unique etiology, but that could benefit from closer follow-up. The drawback of this sequential approach is the high number of false positives (23.6%, n=312). Rather than immediate referral of these cases, we propose that children who pass WHOS and POx, but fail CliML, should have regular follow ups by the CHW as per the WHO guidelines for postnatal care (days 3, 7, 14 and 42 days of life)[25]. During these follow ups, the CHW can use WHOS and POx as tools to re-assess the neonate and refer to a care facility if deterioration occurs. As seen in the simplified antibiotic therapy trial, home based follow up by CHWs at assigned days during the first 2 week of life, helped with timely identification of sick neonates in the community[26]. Further, the mother can also be counseled regarding danger signs in the neonate and the need to seek help immediately[27]. These approaches may be an effective strategy for task sharing and optimizing resource utilization where triage can be done at the community level by frontline care providers without overburdening the health system with unnecessary referrals[28]. This proposed data-driven and staged approach increases the sensitivity, while keeping the false positives manageable in the healthcare setting.

There are some limitations of the current study. The proposed clinical ML model needs to be externally validated in a larger population to assess its performance, given the difference between train and test performance. Additionally, the AllML model should be further explored and trained with larger datasets to assess its value as compared to the proposed sequential approach. The complementary value of using the actual values of hand and foot oxygen saturation together with heart rate, instead of the pass/fail information from the POx may provide important information. After enrollment in the study, 73/1955 children died before any screening was performed, which may indicate a selection bias and may contribute towards the relatively low NMR (12.7/1000 livebirths) seen in this study. Also, the current model includes clinical parameters

Confidential: For Review Only

such as gestational age, which was calculated based on the self-reported date of last menstrual period which is known to have low reliability. Furthermore, POx was performed using one particular device only. Future research would require external validation of the sequential decision-making approach using the ML model in a community-based setting in improving detection of ARN.

Conclusion:

In this study, the value of different screening strategies to detect ARN was assessed. While assessing WHOS and performing POx are highly specific, adding an ML model based on clinical (maternal, neonatal and pregnancy-related) parameters could increase sensitivity. The increase in false-positives would be easily managed if a staged, task sharing approach involving community-based triage, close follow-up and early referral if needed by CHWs, is adopted. This model is also in alignment with the framework presented to decrease the global burden of childhood mortality due to non-communicable diseases.

Confidential: For Review Only

Acknowledgements:

We would like to thank the study staff and the study participants for their contribution in this work.

Data sharing:

Individual participant de-identified data will be made available upon request to the study authors 12 months after publication of this manuscript.

Patient and public involvement: Patients and/or the public were not involved in the design, or conduct, or reporting, or dissemination plans of this research.

Funding support: This work has been funded by the Islamic Development Bank (10/HS/ 650 /441), the Bill and Melinda Gates Foundation (INV-021528), and the Fundació La Marató de TV3 (Ref 202016-30-31).

Contributors: All authors approved the final draft of the manuscript. The corresponding authors attests that all listed authors meet authorship criteria and that no others meeting the criteria have been omitted.

Competing interests: None declared

Confidential: For Review Only

References:

1. NIPS I. Pakistan demographic and health survey 2017-18. Islamabad, Pakistan, and Rockville, Maryland, USA: NIPS, ICF. 2019.
2. Hug L, Alexander M, You D, Alkema L, for Child UI-aG. National, regional, and global levels and trends in neonatal mortality between 1990 and 2017, with scenario-based projections to 2030: a systematic analysis. The Lancet Global Health. 2019;7(6):e710-e20.
3. Therrell BL, Padilla CD, Loeber JG, Kneisser I, Saadallah A, Borrajo GJ, et al., editors. Current status of newborn screening worldwide: 2015. Seminars in perinatology; 2015: Elsevier.
4. Watson MS, Lloyd-Puryear MA, Howell RR. The Progress and Future of US Newborn Screening. International Journal of Neonatal Screening. 2022;8(3):41.
5. Plana MN, Zamora J, Suresh G, Fernandez-Pineda L, Thangaratinam S, Ewer AK. Pulse oximetry screening for critical congenital heart defects. Cochrane Database of systematic reviews. 2018(3).
6. Oster ME, Aucott SW, Glidewell J, Hackell J, Kochilas L, Martin GR, et al. Lessons learned from newborn screening for critical congenital heart defects. Pediatrics. 2016;137(5).
7. Jawin V, Ang H-L, Omar A, Thong M-K. Beyond critical congenital heart disease: newborn screening using pulse oximetry for neonatal sepsis and respiratory diseases in a middle-income country. PloS one. 2015;10(9):e0137580.
8. Williams KB, Horst M, Hollinger EA, Freedman J, Demczko MM, Chowdhury D. Newborn Pulse Oximetry for Infants Born Out-of-Hospital. Pediatrics. 2021;148(4).
9. Emdin CA, Mir F, Sultana S, Kazi A, Zaidi AK, Dimitris MC, et al. Utility and feasibility of integrating pulse oximetry into the routine assessment of young infants at primary care clinics in Karachi, Pakistan: a cross-sectional study. BMC pediatrics. 2015;15(1):1-11.
10. Group YICSS. Clinical signs that predict severe illness in children under age 2 months: a multicentre study. The Lancet. 2008;371(9607):135-42.
11. Ilyas M, Naeem K, Fatima U, Nisar MI, Kazi AM, Jehan F, et al. Profile: health and demographic surveillance system in peri-urban areas of Karachi, Pakistan. Gates Open Research. 2018;2(2):2.
12. Agadoorappa P, Oddie S, Pawson N, Sheridon E. Do pakistani babies have more congenital heart defects? Preliminary findings from our birth cohort study. Archives of Disease in Childhood. 2011;96(Suppl 1):A35-A.
13. Martin GR, Ewer AK, Gaviglio A, Hom LA, Saarinen A, Sontag M, et al. Updated strategies for pulse oximetry screening for critical congenital heart disease. Pediatrics. 2020;146(1).
14. Nichols EK, Byass P, Chandramohan D, Clark SJ, Flaxman AD, Jakob R, et al. The WHO 2016 verbal autopsy instrument: an international standard suitable for automated analysis by InterVA, InSilicoVA, and Tariff 2.0. PLoS medicine. 2018;15(1):e1002486.
15. CDC. Critical Congenital Heart Defects 2022 [cited 2022 May 6]. Available from: https://www.cdc.gov/ncbddd/heartdefects/cchd-facts.html.
16. Prabhakaran D, Anand S, Watkins D, Gaziano T, Wu Y, Mbanya JC, et al. Cardiovascular, respiratory, and related disorders: key messages from Disease Control Priorities. The Lancet. 2018;391(10126):1224-36.
17. Chen T, Guestrin C, editors. Xgboost: A scalable tree boosting system. Proceedings of the 22nd acm sigkdd international conference on knowledge discovery and data mining; 2016.

Confidential: For Review Only

18. Lundberg SM, Lee S-I. A unified approach to interpreting model predictions. Advances in neural information processing systems. 2017;30.
19. Padilla CD, Krotoski D, Therrell Jr BL, editors. Newborn screening progress in developing countries—overcoming internal barriers. Seminars in perinatology; 2010: Elsevier.
20. Hasan BS, Bhatti A, Mohsin S, Barach P, Ahmed E, Ali S, et al. Recommendations for developing effective and safe paediatric and congenital heart disease services in low-income and middle-income countries: a public health framework. BMJ Global Health. 2023;8(5):e012049.
21. Ewer AK. Pulse oximetry screening for critical congenital heart defects in newborn infants: should it be routine? Archives of Disease in Childhood-Fetal and Neonatal Edition. 2014;99(1):F93-F5.
22. McCollum ED, Ginsburg AS. Outpatient management of children with World Health Organization chest indrawing pneumonia: implementation risks and proposed solutions. Clinical Infectious Diseases. 2017;65(9):1560-4.
23. Ng B, Hokanson J. Missed congenital heart disease in neonates. Congenital heart disease. 2010;5(3):292-6.
24. Liske MR, Greeley CS, Law DJ, Reich JD, Morrow WR, Baldwin HS, et al. Report of the Tennessee task force on screening newborn infants for critical congenital heart disease. Pediatrics. 2006;118(4):e1250-e6.
25. Organization WH. WHO recommendations on postnatal care of the mother and newborn: World Health Organization; 2014.
26. Mir F, Nisar I, Tikmani SS, Baloch B, Shakoor S, Jehan F, et al. Simplified antibiotic regimens for treatment of clinical severe infection in the outpatient setting when referral is not possible for young infants in Pakistan (Simplified Antibiotic Therapy Trial [SATT]): a randomised, open-label, equivalence trial. The Lancet Global Health. 2017;5(2):e177-e85.
27. Rahman AE, Herrera S, Rubayet S, Banik G, Hasan R, Ahsan Z, et al. Managing possible serious bacterial infection of young infants where referral is not possible: Lessons from the early implementation experience in Kushtia District learning laboratory, Bangladesh. PloS one. 2020;15(5):e0232675.

Confidential: For Review Only

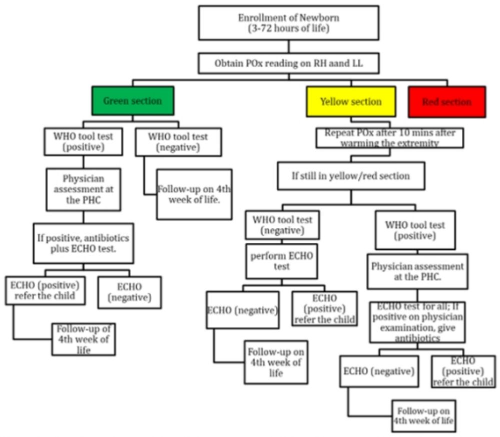

*RH – right hand, LL – lower limb, Pox – pulse oximeter screening, WHO – WHO signs, ECHO – echocardiography, PHC– primary health center. Any child who failed pulse oximeter screening and/or WHO signs by the community health worker at the home assessment was recommended an ECHO

338x190mm (96 x 96 DPI)

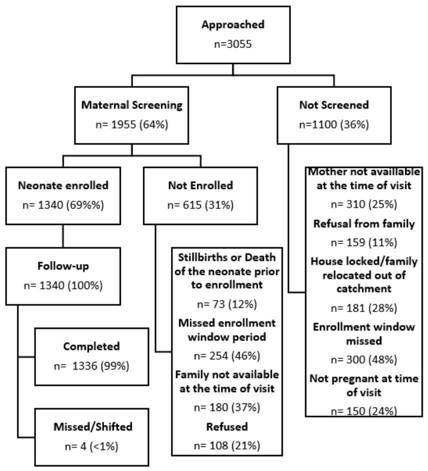

338x190mm (96 x 96 DPI)

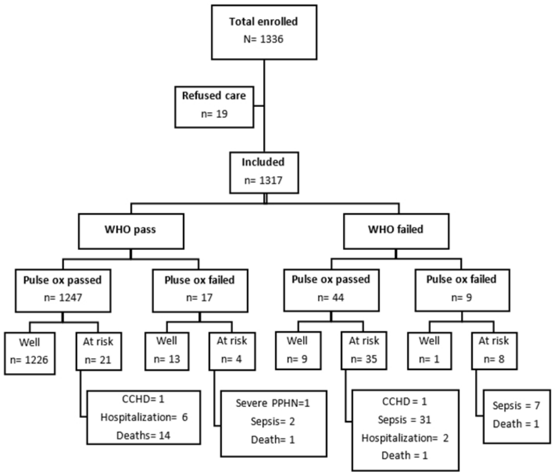

338x190mm (96 x 96 DPI)

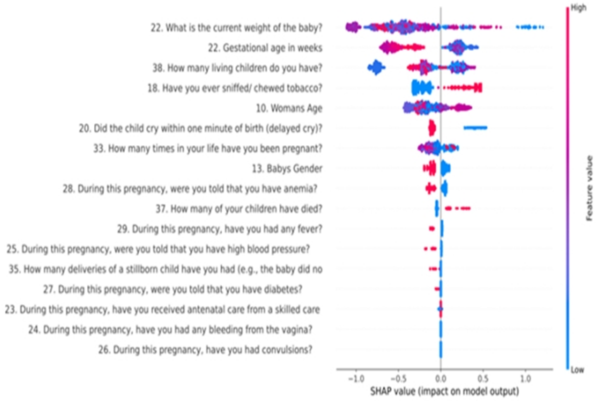

338x190mm (96 x 96 DPI)

SUPPLEMENTARY MATERIAL

Figure S1: CHD screening table using pulse oximetry value on right hand and either foot

Right Hand	Either Foot											
100	100	99	98	97	96	95	94	93	92	91	90	<90
99	100	99	98	97	96	95	94	93	92	91	90	<90
98	100	99	98	97	96	95	94	93	92	91	90	<90
97	100	99	98	97	96	95	94	93	92	91	90	<90
96	100	99	98	97	96	95	94	93	92	91	90	<90
95	100	99	98	97	96	95	94	93	92	91	90	<90
94	100	99	98	97	96	95	94	93	92	91	90	<90
93	100	99	98	97	96	95	94	93	92	91	90	<90
92	100	99	98	97	96	95	94	93	92	91	90	<90
91	100	99	98	97	96	95	94	93	92	91	90	<90
90	100	99	98	97	96	95	94	93	92	91	90	<90
<90	100	99	98	97	96	95	94	93	92	91	90	<90

r Review Only

Table S1: For AllML, the following clinical variables were used:

Group	Variable
Maternal characteristics	Age
	Years of education
	Smoking
	Sniffing/chewing of tobacco
	Chewing of betel nut
	Cardiac disease
	Diabetes
	Thyroid disease
	Any type of cancer
	Epilepsy
Obstetric History	Number of pregnancies
	Number of pregnancies ended before 7 months
	Number of stillborn
	Number of deliveries
	Number of live deliveries
	Number of children died during delivery
	Number of living children
	Number of children died
	Number of children with congenital heart disease
	Number of children with any heart surgery
Current pregnancy characteristics	Received antenatal care from a skilled care
	Vaginal bleeding
	High blood pressure
	Convulsions
	Diabetes
	Anemia
	Fever
	Blood transfusion
	Overnight admission to a health facility
Neonatal characteristics	Gestational age
	Delayed cry
	Weight
	Gender
	Place of birth
	Mode of delivery

Confidential: For Review Only

Table S2: Details of the study participants who died at four weeks of age

S.No	Mode of delivery	Place of delivery	GA at time of delivery (weeks)	Birth weight (kg)	Age at time of enrollment (hours)	WHO Signs	Pulse Oxi-meter	ECHO done	Age at death (in days)	Place of death	Cause of death
**1**	SVD	Clinic	32	2.53	43	Pass	Pass	No	8	Hospital	Neonatal sepsis
**2**	SVD	Hospital	40	2.59	36	Pass	Pass	No	19	Home	Neonatal sepsis
**3**	SVD	Home	40	1.92	6	Fail	Fail	Yes*	13	Home	Neonatal sepsis
**4**	SVD	Hospital	25	1.19	49	Pass	Pass	No	16	Home	Pre-term related complication
**5**	SVD	Clinic	38	2.00	11	Fail	Pass	No (refused)	6	Hospital	Neonatal sepsis
**6**	SVD	Hospital	35	1.92	34	Pass	Pass	No	3	Home	Not determined after VA
**7**	SVD	Hospital	40	2.86	69	Fail	Pass	No (refused)	3	Hospital	Neonatal sepsis
**8**	SVD	Clinic	38	2.96	58	Pass	Pass	No	6	Hospital	Neonatal tetanus
**9**	SVD	Hospital	33	2.34	78	Pass	Pass	No	30	Home	Not determined after VA
**10**	SVD	Clinic	40	4.21	21	Pass	Pass	No	5	Home	Not determined after VA
**11**	SVD	Home	34	1.86	37	Pass	Pass	No	9	Home	Not determined after VA
**12**	SVD	Home	39	2.76	11	Pass	Pass	No	11	Home	Neonatal sepsis
**13**	SVD	Home	51	2.08	31	Pass	Pass	No	28	Hospital	Neonatal sepsis

**14**	Cesarean section	Hospital	36	3.29	96	Pass	Pass	No	20	Home	Not determined after VA
**15**	SVD	Home	40	2.73	18	Pass	Pass	No	26	Home	Neonatal sepsis
**16**	SVD	Hospital	39	2.56	67	Pass	Fail	Yes^	21	Home	Neonatal pneumonia
**17**	Cesarean section	Hospital	36	2.56	94	Pass	Pass	No	18	Home	Neonatal sepsis

*Severe tricuspid regurgitation, ^patent foramen ovale – both ECHOs were performed on the same day of assessment; VA – verbal autopsy;

Confidential: For Review Only

Table S3: Sensitivity and specificity of the pulse oximeter in detecting CCHD or neonatal sepsis

	**For CCHD detection (n=1317)**	**For detection of neonatal sepsis (n=1317)**
True positives (n)	0	9
True negatives (n)	1289	1259
False positives (n)	26	17
False negatives (n)	2	32
Sensitivity (95% CI) (%)	0 (0-84.2)	21.9 (10.6-37.6)
Specificity (95% CI) (%)	98 (97.1-98.7)	98.6 (97.9-99.2)

Confidential: For Review Only

Table S4: Performance metrics of the different machine learning models developed on the clinical, WHO signs and pulse oximetry data

	POx		WHO		POx | WHO		CliML		SeqML		AllML	
	Train	Test	Train	Test	Train	Test	Train	Test	Train	Test	Train	Test
**TP**	7	5	27	17	29	19	37	12	40	23	37	17
**FP**	10	4	5	5	14	9	153	118	165	124	21	15
**FN**	34	22	14	10	12	8	4	15	1	4	4	10
**TN**	739	496	744	495	735	491	596	382	584	376	728	485
**SE**	17	19	66	63	71	70	90	44	98	85	90	63
**SP**	99	99	99	99	98	98	80	76	78	75	97	97
**AUC**	0.58	0.59	0.83	0.81	0.84	0.84	0.85	0.60	0.88	0.80	0.94	0.80
**F1**	0.24	0.28	0.74	0.69	0.69	0.69	0.32	0.15	0.33	0.26	0.75	0.58
**PR**	0.41	0.56	0.84	0.77	0.67	0.68	0.19	0.09	0.20	0.16	0.64	0.53

POx: pulse-oximetry alone; WHO: WHO signs alone; POx | WHO: POx or WHO signs; CliML: ML model for clinical data only; SeqML: sequential use of POx, WHO and CliML; AllML: ML model for the clinical data enriched with WHI signs outcome and POx values (saturations of hand and toe and heart rate); Train: Results for the training set; Test: results for the test set; TP: True Positives; FP: False Positives; FN: False Negatives; TN: True Negatives; SE: Sensitivity; SP: Specificity; AUC: Area under the Curve; F1: F1 score; PR: Precision)

Confidential: For Review Only

Figure S2: SHAP analysis for the ML model based on the clinical variables.

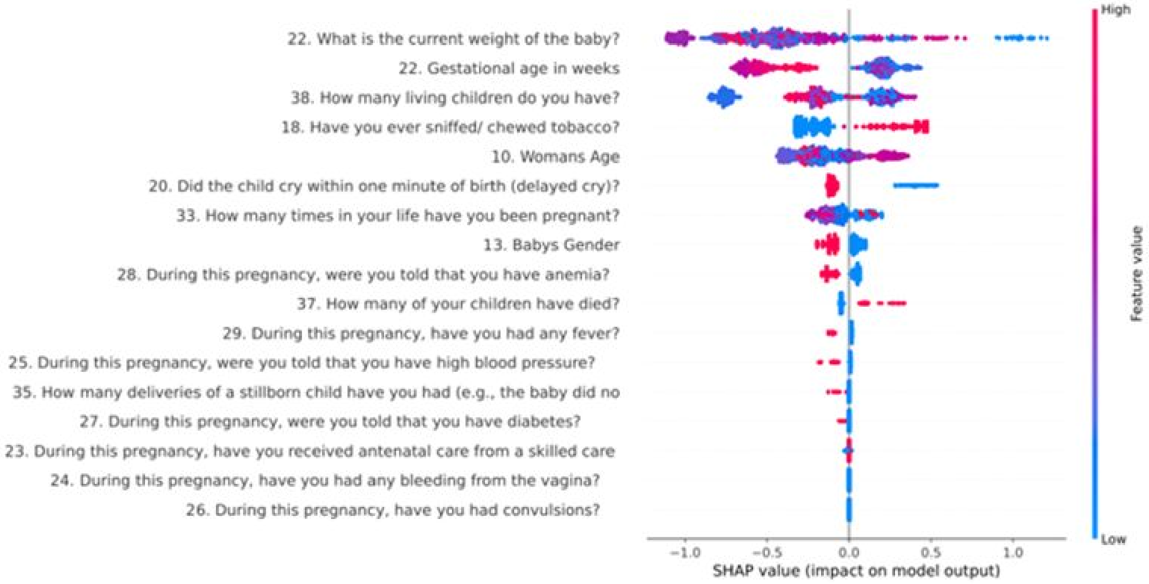

For Review Only

Figure S3: ROC analysis for the ML based on clinical variables as well as the sequential use of WHOS, POx and clinical ML

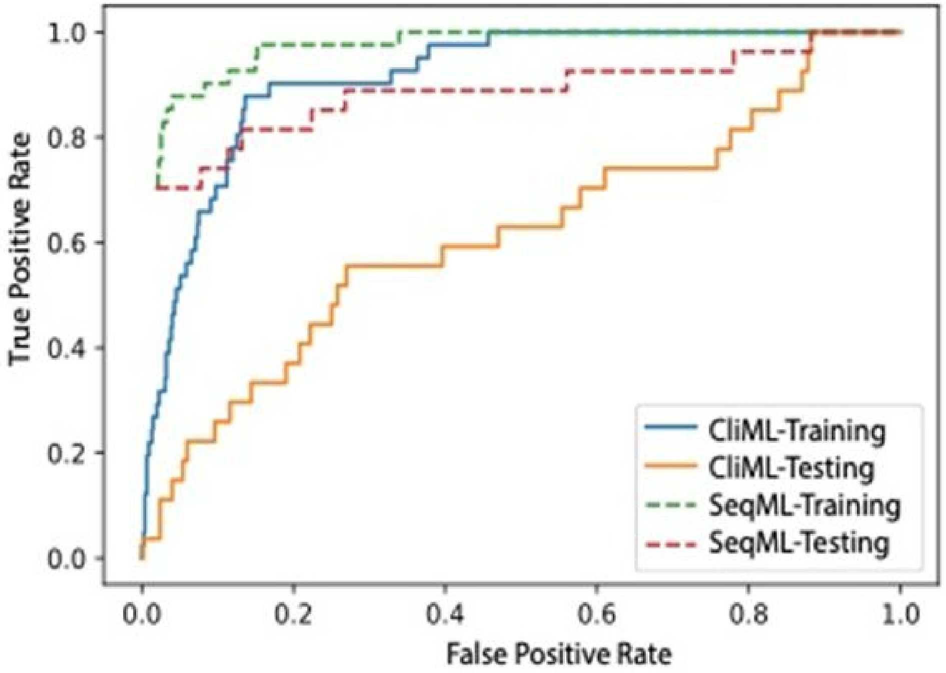

For Peer Review Only

BMJ Paediatrics Open

A sequential approach of classical screening enriched with machine learning improves neonatal wellness screening in low resource settings: a prospective cohort study

Journal:	*BMJ Paediatrics Open*
Manuscript ID	bmjpo-2023-002134.R1
Article Type:	Original research
Date Submitted by the Author:	07-Sep-2023
Complete List of Authors:	Hasan, Babar; Sindh Institute of Urology and Transplantation, Department of Pediatrics and Child Health<br>Hoodbhoy, Zahra; Aga Khan University, Department of Pediatrics and Child Health<br>Khan, Amna; The Aga Khan University, Department of Pediatrics and Child Health<br>Nogueira, Mariana; IDIBAPS<br>Bijnens, Bart; IDIBAPS; ICREA<br>Chowdhury, Devyani; The Children's Hospital of Philadelphia
Keywords:	Screening, Neonatology

SCHOLARONE™
Manuscripts

I, the Submitting Author has the right to grant and does grant on behalf of all authors of the Work (as defined in the below author licence), an exclusive licence and/or a non-exclusive licence for contributions from authors who are: i) UK Crown employees; ii) where BMJ has agreed a CC-BY licence shall apply, and/or iii) in accordance with the terms applicable for US Federal Government officers or employees acting as part of their official duties; on a worldwide, perpetual, irrevocable, royalty-free basis to BMJ Publishing Group Ltd ("BMJ") its licensees and where the relevant Journal is co-owned by BMJ to the co-owners of the Journal, to publish the Work in this journal and any other BMJ products and to exploit all rights, as set out in our licence.

The Submitting Author accepts and understands that any supply made under these terms is made by BMJ to the Submitting Author unless you are acting as an employee on behalf of your employer or a postgraduate student of an affiliated institution which is paying any applicable article publishing charge ("APC") for Open Access articles. Where the Submitting Author wishes to make the Work available on an Open Access basis (and intends to pay the relevant APC), the terms of reuse of such Open Access shall be governed by a Creative Commons licence – details of these licences and which Creative Commons licence will apply to this Work are set out in our licence referred to above.

Other than as permitted in any relevant BMJ Author's Self Archiving Policies, I confirm this Work has not been accepted for publication elsewhere, is not being considered for publication elsewhere and does not duplicate material already published. I confirm all authors consent to publication of this Work and authorise the granting of this licence.

Confidential: For Review Only

Title: A sequential approach of classical screening enriched with machine learning improves neonatal wellness screening in low resource settings: a prospective cohort study

Authors:

Babar S. Hasan*[1] MBBS, Zahra Hoodbhoy*[2] PhD, Amna Khan[2] BA, Mariana Nogueira[3] PhD, Bart Bijnens PhD[3,4], Devyani Chowdhury[5,6] MD

*Shared first authorship

[#]Shared senior authorship

[1]Department of Pediatrics and Child Health, Stadium Road, The Aga Khan University, Karachi, Pakistan relocated to Sindh Institute of Urology and Transplantation (SIUT), Karachi, Pakistan

[2]Department of Pediatrics and Child Health, Stadium Road, The Aga Khan University, Karachi, Pakistan

[3]IDIBAPS (August Pi i Sunyer Biomedical Research Institute), Barcelona, Spain

[4]ICREA, Barcelona, Spain

[5]Cardiology Care for Children, Pennsylvania, USA,

[6]Nemours Cardiac Center, Wilmington, DE

Corresponding author:

Babar Hasan
Professor and Chair
Division of Cardiothoracic Sciences
Sindh Institute of Urology and Transplantation (SIUT)
Karachi, Pakistan
Tel No: +92-3362559473
Email address: drbabarhasan@yahoo.com

Confidential: For Review Only

Abstract:

Introduction: Timely identification of at-risk neonates (ARN) in the community is essential to reduce mortality in low resource settings. Tools such as AAP pulse-oximetry (POx) and WHO Young Infants Clinical Signs (WHOS) have high specificity but low sensitivity to identify at risk neonates (ARN). Our aim was assessing the value of POx and WHOS independently, in combination, and with machine learning (ML) from clinical features, to detect ARN in a low- to middle-income country (LMIC)

Methods: This prospective cohort study was conducted in a peri-urban community in Pakistan. Eligible live births were screened using WHOS and POx along with clinical information regarding pregnancy and delivery. The enrolled neonates were followed for 4 weeks of life to assess the vital status. The predictive value to identify ARN, of POx, WHOS and an ML model using maternal and neonatal clinical features was assessed.

Results: Of 1336 neonates, 68 (5%) had adverse outcomes, i.e. sepsis (n=40, 59%), critical congenital heart disease (n=2, 3%), severe persistent pulmonary hypertension (n=1), hospitalization (n=8, 12%) and death (n=17, 25%) assessed at 4 weeks of life. Specificity of POx and WHOS to independently identify ARN was 99%, with sensitivity of 19% and 63% respectively. Combining both improved SE to 70%, keeping SP at 98%. An ML model using clinical variables had 44% SE and 76% SP. A staged assessment, where WHOs, POx and ML are sequentially used for triage increased SE to 85%, keeping SP 75%. Using ML (when WHO and POx negative) for community follow-up detected the majority of ARN.

Conclusion: Classical screening combined with ML, can help maximizing identifying ARN and could be embedded in low-resource clinical settings, thereby improving outcome. Sequential use

Confidential: For Review Only

of classical assessment and clinical ML identifies the most at-risk neonates in the community, still optimizing follow-up clinical care.

Keywords: neonatal wellness; screening; community health workers; machine learning

Confidential: For Review Only

Introduction:

With a neonatal mortality rate (NMR) of 42 per 1000 live births, Pakistan accounts for 7% of all global neonatal deaths and ranks 3rd on the list among countries with highest NMR.[1] The top four causes of neonatal deaths are pre-maturity, birth asphyxia, sepsis and congenital anomalies, most of which are congenital heart diseases (CHD).[2]

Newborn screening at few hours of life, for identification of at-risk newborns (ARN), has been proposed as an effective tool to reduce NMR.[3] In high-income countries (HICs), a combination of antenatal ultrasound and newborn screening enables early detection, diagnosis, and intervention to prevent death or disability. Newborn screening, using a few drops of blood for genetic, metabolic and endocrine disorders, and American Academy of Pediatrics (AAP) pulse oximetry (POx) for critical congenital heart defects (CCHDs) are widely implemented in HICs.[4]

A recent systematic review by Plana et al reported that the overall sensitivity of pulse oximetry for the detection of CCHD was 76.3% (95% CI 69.5 to 82.0) (low certainty of the evidence) while the specificity was 99.9% (95% CI 99.7 to 99.9) (high certainty of the evidence).[5] While POx is a highly specific tool to detect CCHD, it will detect other non-cardiac conditions associated with hypoxia, such as neonatal sepsis, hypothermia and persistent pulmonary hypertension,[6, 7] where it has demonstrated a high specificity (>99.9%) but low sensitivity (42%).[8] POx thus has shown a broader utility as a newborn wellness screen, with a good feasibility and acceptability for screening newborns in primary care centers in low middle income countries (LMICs).[9]

A complementary newborn wellness screening tool widely used in LMICs, is the WHO Young Infants Clinical Signs Study Group (WHOS), which identifies seven signs including fever ≥ 38°C, hypothermia (temperature < 35°C), convulsions, lethargy, poor feeding, chest indrawing

Confidential: For Review Only

and tachypnea (respiratory rate > 60 breaths per minute) and has a sensitivity of 74% and specificity of 79% to predict severe illness.[10] Though WHOS and POx have shown only modest sensitivity, these are simple and highly specific screening tools which may be used to identify newborn conditions contributing to NMR in low resource settings.

Given their moderate sensitivity, there remains a need to optimize the performance of these tools and to develop improved strategies by including the use of a wider set of clinical parameters for community-based triage of ARN.[10] The aim of this study was to assess the value of POx and WHOS, used on their own, in combination, and enriched with machine learning (ML) from clinical features, to develop a screening tool for community health workers (CHWs) to identify ARN.

Confidential: For Review Only

Methods:

Study design and participants:

This was an ethics committee approved (Aga Khan University ERC number: 2019-0551-4976) single group prospective cohort study conducted at Ibrahim Hyderi (IH), a peri-urban settlement of approximately 100,000 individuals on the south-east of Karachi, Pakistan, from October 1, 2020 to March 31, 2022. The main occupation of individuals in this area is fishing with 40% below the poverty line.[11] The NMR at this site ranges between 40-45 per 1000 live births.[11] The eligibility criterion was a single live birth where the mother planned to stay in the study catchment area for at least four weeks after delivery.

Sample size estimation:

The sample size was estimated based on the primary outcomes of this study. Neonatal sepsis has been reported to be as high as 14%[2] while the prevalence of CHD in Pakistan is stated to be around 0.9%.[12] With a 95% confidence interval, power of 80% and absolute precision of 0.6%, a frequency of 0.9% generated the maximum sample size of 951 neonates. To account for 5% loss to follow up, the sample size was inflated to 1000 neonates.

Procedures:

The Department of Pediatrics and Child Health at The Aga Khan University (AKU) maintains a demographic and health surveillance system at this site whereby all households are enumerated. Leveraging the surveillance data for identification of pregnant women, we used a nonprobability convenience sampling approach where trained CHWs visited these households to screen eligible women and obtained written informed consent for the participation of their newborn in the research study. All live births registered within 72 hours, where clinical information about

Confidential: For Review Only

the pregnancy and delivery were collected (refer to table S1) while danger signs were assessed using WHOS[10] along with POx (using the Masimo Rad-8 pulse oximeter).

The oxygen saturation (SpO2) was measured on the right hand and foot (30 seconds each) once the device's signal quality index showed stable green bars for >10 seconds. The SpO2 readings were recorded and categorized into green, yellow and red zone as per AAP recommendations (refer to figure 1 and Figure S1).[13] Neonates who had a positive WHOS or fell in the yellow or red zone for POx, were referred for further evaluation and echocardiography with follow up by a physician at the primary health care center (PHC). As per PHC protocol, all clinically sick neonates who were prescribed antibiotics were followed daily for the next 7 days. All neonates were also followed up at four weeks of life to assess the vital status of the child, and hospitalizations, if any, during this period. For all neonatal deaths, a verbal autopsy was performed using the WHO 2016 verbal autopsy instrument by trained research staff[14] and coded by a physician to ascertain cause of death.

Outcomes:

The primary outcome of this study was the identification of ARN, defined as:

- Early neonatal sepsis – Confirmed by a physician after referral based on WHOS or other clinical indications.[10]
- Critical CHD (CCHDs) – Confirmed by echocardiography (transposition of the great arteries, double outlet right ventricle, complex single ventricle physiology, Tetralogy of Fallot, truncus arterious.[15]
- Other cardiac conditions, such as severe persistent pulmonary hypertension (PPHN) (bi-directional patent ductus arteriosus (PDA) and right ventricular dysfunction.)
- Hospitalization/death within the first four weeks of life.

Confidential: For Review Only

Neonates with isolated non-hemodynamically significant CHDs, like small ventricular septal defects (VSD) and atrial septal defects (ASD),[16] were not considered as ARN.

Statistical analysis and Machine Learning:

Conventional data analysis was performed using STATA 16.4 bit. Data was reported as percentages and means +/- standard deviation where appropriate.

For the ML, we used the XGBoost implementation of gradient boosted decision trees.[17] For the first model (AllML), an extensive set of clinical variables (table S1), the measurements obtained from POx, and the outcome of WHOS (pass/fail) was used; for the second model (CliML), a smaller set of clinical variables selected based on a combination of prior expert knowledge on relevant features and the results of a model explainer, ranking variables according to importance for outcome prediction in AllML. We also used the SHapley Additive exPlanations (SHAP)[18] approach to understand the outputs of the ML models. The models were trained on 60% of the data and using 3-fold cross validation while 40% were kept as test set. During the training stage, a grid search was performed to tune model hyperparameters, and those yielding the best cross-validation F1-score were selected. The F1-score and other performance metrics (sensitivity, specificity, precision, the Receiver Operating Characteristic (ROC) and respective Area Under the Curve (AUC) were then computed for the full training and the testing set.

Finally, a sequential use (SeqML) of WHOS and if negative followed by POx and if that too is negative then CliML was assessed. A ROC curve was constructed using a moving threshold on the probability provided by CliML and adding this to the (fixed) sensitivities and specificities of POx after WHOS.

Confidential: For Review Only

Results:

During the study period, 1336 neonates were enrolled from the community (figure 2). Maternal and neonatal characteristics of the study population are described in Table 1. The mean maternal age was 27+/- 5.1 years, 46% (n=618) did not have any formal education, 92%(n=933) had received at least one antenatal visit during the current pregnancy and, one third (n=397) delivered at home.

Table 1: Baseline characteristics of mothers and neonates enrolled in the study

**Maternal characteristics (N=1336)**	
Maternal age (in years), mean ± SD	27+/-5.1
Literacy rate, *n (%)*	618 (46)
Unemployed, *n (%)*	1275 (95)
Number of pregnancies, Median IQR	3 (1-14)
Number of alive children, Median IQR	2 (0-10)
Received antenatal care in the current pregnancy, *n (%)*	1248 (93)
**Neonatal characteristics (N=1336)**	
GA at delivery (in weeks), mean ± SD	39+/-4.5
Preterm	313 (23)
Term	1023 (77)
Place of birth, *n (%)*	
Facility	939 (70)
Home	397 (30)
Mode of delivery, *n (%)*	
Spontaneous vaginal delivery	1015 (76)
Assisted vaginal delivery	3 (<1)
Cesarean section	318 (24)
Gender, *n (%)*	
Female	646 (48)
Male	690 (52)

Confidential: For Review Only

Hours at time of enrollment, Median IQR<br>< 24 hours<br>24-48 hours<br>48 -72 hours<br>>72 hours	48.2 (0-141)<br>256 (19)<br>462 (34.5)<br>389 (29.1)<br>229 (17.1)
Weight at enrollment in kg, Median IQR	2.84 (1.19-5.72)
Difference in hours between pulse oximeter screening and echocardiography<br>< 24 hours<br>24-72 hours<br>>72 hours	 <br>42 (60)<br>14 (20)<br>14 (20)

For the final outcome at four weeks follow up, neonates were labeled as *well* or *ARN*. There were 19 neonates who were excluded from the analysis due to refusal for follow up. We identified 68/1317 (5.1%) ARN. These included 40 cases (59%) of neonatal sepsis, 2 cases (3%) of CCHD (both Tetralogies of Fallot, one also with sepsis), 1 case (1.5%) of severe PPHN (bi-directional PDA with right ventricular dysfunction), 8 (11.5%) hospitalizations and 17 deaths (25%). The detailed characteristics of the study participants who died are described in Table S2. Of the neonatal deaths (n=17), 76% (n=13) passed both WHOS and pulse oximeter screening. Five of these (30%) had the screening performed at less than 24 hours of life. Five were early neonatal deaths with approximately 2.5 days between enrollment and death while for the 12 late neonatal deaths, the median difference between enrollment and death was 16.5 days (IQR 6-26.5).

The distribution of the neonates according to WHOS and POx screening is shown in figure 3 with more details in table S3.

ARN screening performance

1317 neonates were included in the predictive modeling to assess ARN. The training set (60%) consisted of 790 neonates while the test set included 527. Table S4 summarizes all results.

Confidential: For Review Only

As expected, WHOS (absent/present) and POx (pass/fail) showed a high specificity, but only moderate sensitivity to detect ARN. Combining both improved overall sensitivity as compared to the singular tests, while preserving specificity. When comparing the results in training and test set, they showed similar performance, confirming their stable performance in this population.

The ML model based on the clinical variables (CliML) showed good sensitivity/specificity on training, but much worse performance during testing, indicating overfitting.

AllML, trained on all assessed information at once, showed excellent performance at training, but reduced sensitivity in the test set indicating overfitting,. The variables that contributed most to the decision included maternal features such as tobacco use, previous pregnancies, child deaths and pregnancy complication, as well as neonatal features, such as gender, gestational age at delivery and birthweight as indicated by the SHAP analysis (figure S2).

To find a balance between the high specificity of WHOS and POx and the potentially higher sensitivity of CliML, a sequential testing approach, composed of first assessing WHOS, followed by POx and CliML to further identify ARN was investigated (figure 4). Despite some overfitting during training, SeqML clearly showed increased sensitivity in the test set, as compared to all other approaches. Figure S3 shows the ROC curve for both CliML as well as the sequential approach of SeqML.

Confidential: For Review Only

Discussion:

High NMR in LMIC is an important public health problem that can be addressed by timely detection of ARN. It is important to identify a community based implementation framework where neonates can be appropriately followed by confirmatory testing and affordable management pathways.[19]

This study showed that a sequentially deployed strategy for identification of ARN, can outperform the traditional approach of using only POx or WHO signs. The model identifies a maximal number of ARN and can be implemented in a staged manner where those failing WHOS and POx can be referred sooner to a higher-level care facility, while intermediate risk neonates detected by the SeqML are monitored at home by a CHW.

WHOS has modest sensitivity to detect non-infectious etiologies. In the framework presented by the Global Initiative for Children's cardiac Surgery, PO and WHOS are a part of basic infrastructure for timely outpatient screening for cardiac disease.[20] POx is recommended for screening for CCHD and is also able to detect other hypoxia causing non-cardiac conditions.[13, 21] This has led to proposed recommendations of the WHO Integrated Management of Childhood Illnesses (IMCI) guidelines for detecting severe pneumonia to include oxygen saturation of <93% as a point for referral.[22] In this study, POx is highly specific for identifying ARN, but has a poor sensitivity when used stand-alone. Amongst factors that might contribute to this is the timing of the screening as while the ductus arteriosus is still open there is a higher sensitivity to detect life-threatening CCHD.[23, 24] In the current study, there were four cases who were screened > 24 hours after birth, did not have WHOS and passed the POx screening, but died within 7-14 days of birth at home where cause of death could not be ascertained. It may be possible that an underlying cardiac condition, especially left sided lesions, may have been

Confidential: For Review Only

missed due to late neonatal screening. Additionally, the POx screening devices used only provided readings of the saturation values and heart rate and did not have visual feedback on acquired waveforms. While out of the scope of this manuscript, when examining the stored waveforms, it was observed that the quality of tracing might have also contributed to the lower sensitivity.

Besides the outcome of WHOS and POx for screening, the study showed that their combination allows to increase sensitivity while maintaining high specificity, indicating detecting other etiologies associated with ARN. However, the modest sensitivity of the combination of both indicates that not all ARN are identified. CliML provided predictive information, but only at modest sensitivity and specificity. Training a singular ML model (AllML) using all available information at once showed potential but had problems with generalizability given that the performance in the test data was inferior to the training. The sequential screening model (SeqML) where WHOS, a specific test first identifies neonates at risk of sepsis and then identifies etiologies associated with decreased saturations, through POx, and finally identifies those at higher risks using an ML algorithm based on clinical information, was most successful in identifying ARN. This approach optimizes a staged follow-up given that WHOS and POx identify those that clearly need urgent medical attention, while the clinical data that can be embedded and entered in an application on the CHW's mobile phone that identifies an increased risk without unique etiology, but that could benefit from closer follow-up. The drawback of this sequential approach is the high number of false positives (23.6%, n=312). Rather than immediate referral of these cases, we propose that children who pass WHOS and POx, but fail CliML, should have regular follow ups by the CHW as per the WHO guidelines for postnatal care and refer if needed.(days 3, 7, 14 and 42 days of life).[25] During these follow ups, the CHW can use WHOS and POx as tools to reassess the neonate and refer to a care facility if deterioration occurs. As seen in the simplified

Confidential: For Review Only

antibiotic therapy trial, home based follow up by CHWs at assigned days during the first 2 week of life, helped with timely identification of sick neonates.[26] Further, the mother can also be counseled regarding danger signs in the neonate and the need to seek help immediately.[27] These approaches may be an effective strategy for task sharing and optimizing resource utilization s without overburdening the health system with unnecessary referrals.[28] This proposed data-driven and staged approach increases the sensitivity, while keeping the false positives manageable in the healthcare setting.

There are some limitations of the current study. The proposed clinical ML model needs to be externally validated in a larger population to assess its performance, given the difference between train and test performance. Additionally, the AllML model should be further explored and trained with larger datasets to assess its value as compared to the proposed sequential approach. The complementary value of using the actual values of hand and foot oxygen saturation together with heart rate, instead of the pass/fail information from the POx may provide important information. After enrollment in the study, 73/1955 children died before any screening was performed, which may indicate a selection bias and may contribute towards the relatively low NMR (12.7/1000 livebirths) seen in this study. Also, the current model includes clinical parameters such as gestational age, which was calculated based on the self-reported date of last menstrual period which is known to have low reliability. Furthermore, POx was performed using one particular device only. Future research would require external validation of the sequential decision-making approach using the ML model in a community-based setting in improving detection of ARN.

Conclusion:

Confidential: For Review Only

In this study, the value of different screening strategies to detect ARN was assessed. While assessing WHOS and performing POx are highly specific, adding an ML model based on clinical (maternal, neonatal and pregnancy-related) parameters could increase sensitivity. The increase in false-positives would be easily managed if a staged, task sharing approach involving community-based triage, close follow-up and early referral if needed by CHWs, is adopted. This model is also in alignment with the framework presented to decrease the global burden of childhood mortality due to non-communicable diseases.

Confidential: For Review Only

Acknowledgements:

We would like to thank the study staff and the study participants for their contribution in this work.

Data sharing:

Individual participant de-identified data will be made available upon request to the study authors 12 months after publication of this manuscript**.**

Patient and public involvement: Patients and/or the public were not involved in the design, or conduct, or reporting, or dissemination plans of this research.

Funding support: This work has been funded by the Islamic Development Bank (10/HS/ 650 /441), the Bill and Melinda Gates Foundation (INV-021528), and the Fundació La Marató de TV3 (Ref 202016-30-31).

Contributors: All authors approved the final draft of the manuscript. The corresponding authors attests that all listed authors meet authorship criteria and that no others meeting the criteria have been omitted.

Competing interests: None declared

Confidential: For Review Only

References:

1. NIPS I. Pakistan demographic and health survey 2017-18. Islamabad, Pakistan, and Rockville, Maryland, USA: NIPS, ICF. 2019.
2. Hug L, Alexander M, You D, Alkema L, for Child UI-aG. National, regional, and global levels and trends in neonatal mortality between 1990 and 2017, with scenario-based projections to 2030: a systematic analysis. The Lancet Global Health. 2019;7(6):e710-e20.
3. Therrell BL, Padilla CD, Loeber JG, Kneisser I, Saadallah A, Borrajo GJ, et al., editors. Current status of newborn screening worldwide: 2015. Seminars in perinatology; 2015: Elsevier.
4. Watson MS, Lloyd-Puryear MA, Howell RR. The Progress and Future of US Newborn Screening. International Journal of Neonatal Screening. 2022;8(3):41.
5. Plana MN, Zamora J, Suresh G, Fernandez-Pineda L, Thangaratinam S, Ewer AK. Pulse oximetry screening for critical congenital heart defects. Cochrane Database of Systematic Reviews. 2018(3).
6. Oster ME, Aucott SW, Glidewell J, Hackell J, Kochilas L, Martin GR, et al. Lessons learned from newborn screening for critical congenital heart defects. Pediatrics. 2016;137(5).
7. Williams KB, Horst M, Hollinger EA, Freedman J, Demczko MM, Chowdhury D. Newborn Pulse Oximetry for Infants Born Out-of-Hospital. Pediatrics. 2021;148(4).
8. Jawin V, Ang H-L, Omar A, Thong M-K. Beyond critical congenital heart disease: newborn screening using pulse oximetry for neonatal sepsis and respiratory diseases in a middle-income country. PloS one. 2015;10(9):e0137580.
9. Emdin CA, Mir F, Sultana S, Kazi A, Zaidi AK, Dimitris MC, et al. Utility and feasibility of integrating pulse oximetry into the routine assessment of young infants at primary care clinics in Karachi, Pakistan: a cross-sectional study. BMC pediatrics. 2015;15(1):1-11.
10. Group YICSS. Clinical signs that predict severe illness in children under age 2 months: a multicentre study. The Lancet. 2008;371(9607):135-42.
11. Ilyas M, Naeem K, Fatima U, Nisar MI, Kazi AM, Jehan F, et al. Profile: health and demographic surveillance system in peri-urban areas of Karachi, Pakistan. Gates Open Research. 2018;2(2):2.
12. Agadoorappa P, Oddie S, Pawson N, Sheridon E. Do pakistani babies have more congenital heart defects? Preliminary findings from our birth cohort study. Archives of Disease in Childhood. 2011;96(Suppl 1):A35-A.
13. Martin GR, Ewer AK, Gaviglio A, Hom LA, Saarinen A, Sontag M, et al. Updated strategies for pulse oximetry screening for critical congenital heart disease. Pediatrics. 2020;146(1).
14. Nichols EK, Byass P, Chandramohan D, Clark SJ, Flaxman AD, Jakob R, et al. The WHO 2016 verbal autopsy instrument: an international standard suitable for automated analysis by InterVA, InSilicoVA, and Tariff 2.0. PLoS medicine. 2018;15(1):e1002486.
15. CDC. Critical Congenital Heart Defects 2022 [cited 2022 May 6]. Available from: https://www.cdc.gov/ncbddd/heartdefects/cchd-facts.html.
16. Prabhakaran D, Anand S, Watkins D, Gaziano T, Wu Y, Mbanya JC, et al. Cardiovascular, respiratory, and related disorders: key messages from Disease Control Priorities. The Lancet. 2018;391(10126):1224-36.
17. Chen T, Guestrin C, editors. Xgboost: A scalable tree boosting system. Proceedings of the 22nd acm sigkdd international conference on knowledge discovery and data mining; 2016.

Confidential: For Review Only

18. Lundberg SM, Lee S-I. A unified approach to interpreting model predictions. Advances in neural information processing systems. 2017;30.
19. Padilla CD, Krotoski D, Therrell Jr BL, editors. Newborn screening progress in developing countries—overcoming internal barriers. Seminars in perinatology; 2010: Elsevier.
20. Hasan BS, Bhatti A, Mohsin S, Barach P, Ahmed E, Ali S, et al. Recommendations for developing effective and safe paediatric and congenital heart disease services in low-income and middle-income countries: a public health framework. BMJ Global Health. 2023;8(5):e012049.
21. Ewer AK. Pulse oximetry screening for critical congenital heart defects in newborn infants: should it be routine? Archives of Disease in Childhood-Fetal and Neonatal Edition. 2014;99(1):F93-F5.
22. McCollum ED, Ginsburg AS. Outpatient management of children with World Health Organization chest indrawing pneumonia: implementation risks and proposed solutions. Clinical Infectious Diseases. 2017;65(9):1560-4.
23. Ng B, Hokanson J. Missed congenital heart disease in neonates. Congenital heart disease. 2010;5(3):292-6.
24. Liske MR, Greeley CS, Law DJ, Reich JD, Morrow WR, Baldwin HS, et al. Report of the Tennessee task force on screening newborn infants for critical congenital heart disease. Pediatrics. 2006;118(4):e1250-e6.
25. Organization WH. WHO recommendations on postnatal care of the mother and newborn: World Health Organization; 2014.
26. Mir F, Nisar I, Tikmani SS, Baloch B, Shakoor S, Jehan F, et al. Simplified antibiotic regimens for treatment of clinical severe infection in the outpatient setting when referral is not possible for young infants in Pakistan (Simplified Antibiotic Therapy Trial [SATT]): a randomised, open-label, equivalence trial. The Lancet Global Health. 2017;5(2):e177-e85.
27. Rahman AE, Herrera S, Rubayet S, Banik G, Hasan R, Ahsan Z, et al. Managing possible serious bacterial infection of young infants where referral is not possible: Lessons from the early implementation experience in Kushtia District learning laboratory, Bangladesh. PloS one. 2020;15(5):e0232675.

Confidential: For Review Only

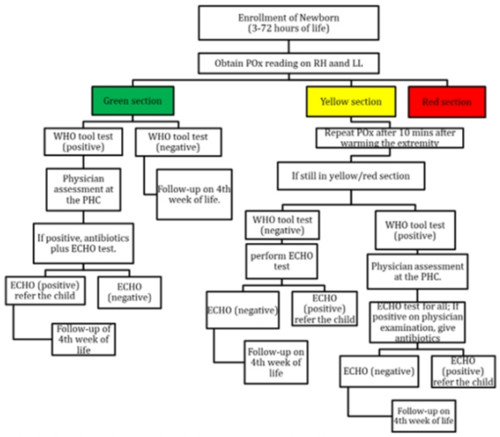

*RH – right hand, LL – lower limb, Pox – pulse oximeter screening, WHO – WHO signs, ECHO – echocardiography, PHC– primary health center. Any child who failed pulse oximeter screening and/or WHO signs by the community health worker at the home assessment was recommended an ECHO

338x190mm (96 x 96 DPI)

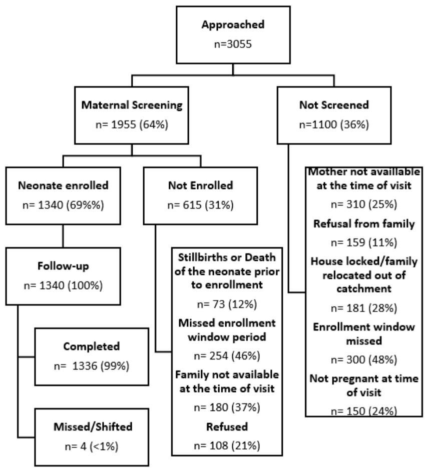

338x190mm (96 x 96 DPI)

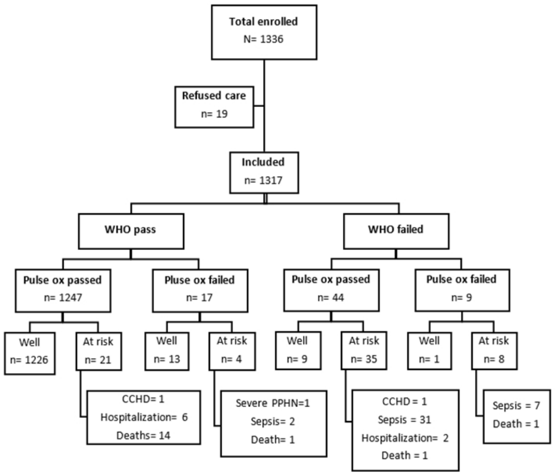

338x190mm (96 x 96 DPI)

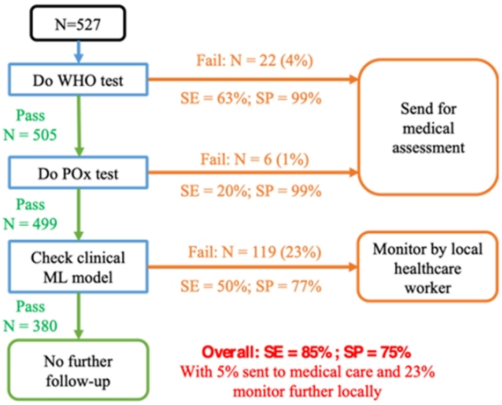

Figure 4

338x190mm (96 x 96 DPI)

SUPPLEMENTARY MATERIAL

Figure S1: CHD screening table using pulse oximetry value on right hand and either foot

Right Hand	Either Foot											
100	100	99	98	97	96	95	94	93	92	91	90	<90
99	100	99	98	97	96	95	94	93	92	91	90	<90
98	100	99	98	97	96	95	94	93	92	91	90	<90
97	100	99	98	97	96	95	94	93	92	91	90	<90
96	100	99	98	97	96	95	94	93	92	91	90	<90
95	100	99	98	97	96	95	94	93	92	91	90	<90
94	100	99	98	97	96	95	94	93	92	91	90	<90
93	100	99	98	97	96	95	94	93	92	91	90	<90
92	100	99	98	97	96	95	94	93	92	91	90	<90
91	100	99	98	97	96	95	94	93	92	91	90	<90
90	100	99	98	97	96	95	94	93	92	91	90	<90
<90	100	99	98	97	96	95	94	93	92	91	90	<90

For Review Only

Table S1: For AllML, the following clinical variables were used:

Group	Variable
Maternal characteristics	Age
	Years of education
	Smoking
	Sniffing/chewing of tobacco
	Chewing of betel nut
	Cardiac disease
	Diabetes
	Thyroid disease
	Any type of cancer
	Epilepsy
Obstetric History	Number of pregnancies
	Number of pregnancies ended before 7 months
	Number of stillborn
	Number of deliveries
	Number of live deliveries
	Number of children died during delivery
	Number of living children
	Number of children died
	Number of children with congenital heart disease
	Number of children with any heart surgery
Current pregnancy characteristics	Received antenatal care from a skilled care
	Vaginal bleeding
	High blood pressure
	Convulsions
	Diabetes
	Anemia
	Fever
	Blood transfusion
	Overnight admission to a health facility
Neonatal characteristics	Gestational age
	Delayed cry
	Weight
	Gender
	Place of birth
	Mode of delivery

Confidential: For Review Only

Table S2: Details of the study participants who died at four weeks of age

S.No	Mode of delivery	Place of delivery	GA at time of delivery (weeks)	Birth weight (kg)	Age at time of enrollment (hours)	WHO Signs	Pulse Oxi-meter	ECHO done	Age at death (in days)	Place of death	Cause of death
**1**	SVD	Clinic	32	2.53	43	Pass	Pass	No	8	Hospital	Neonatal sepsis
**2**	SVD	Hospital	40	2.59	36	Pass	Pass	No	19	Home	Neonatal sepsis
**3**	SVD	Home	40	1.92	6	Fail	Fail	Yes*	13	Home	Neonatal sepsis
**4**	SVD	Hospital	25	1.19	49	Pass	Pass	No	16	Home	Pre-term related complication
**5**	SVD	Clinic	38	2.00	11	Fail	Pass	No (refused)	6	Hospital	Neonatal sepsis
**6**	SVD	Hospital	35	1.92	34	Pass	Pass	No	3	Home	Not determined after VA
**7**	SVD	Hospital	40	2.86	69	Fail	Pass	No (refused)	3	Hospital	Neonatal sepsis
**8**	SVD	Clinic	38	2.96	58	Pass	Pass	No	6	Hospital	Neonatal tetanus
**9**	SVD	Hospital	33	2.34	78	Pass	Pass	No	30	Home	Not determined after VA
**10**	SVD	Clinic	40	4.21	21	Pass	Pass	No	5	Home	Not determined after VA
**11**	SVD	Home	34	1.86	37	Pass	Pass	No	9	Home	Not determined after VA
**12**	SVD	Home	39	2.76	11	Pass	Pass	No	11	Home	Neonatal sepsis
**13**	SVD	Home	51	2.08	31	Pass	Pass	No	28	Hospital	Neonatal sepsis

**14**	Cesarean section	Hospital	36	3.29	96	Pass	Pass	No	20	Home	Not determined after VA
**15**	SVD	Home	40	2.73	18	Pass	Pass	No	26	Home	Neonatal sepsis
**16**	SVD	Hospital	39	2.56	67	Pass	Fail	Yes^	21	Home	Neonatal pneumonia
**17**	Cesarean section	Hospital	36	2.56	94	Pass	Pass	No	18	Home	Neonatal sepsis

*Severe tricuspid regurgitation, ^patent foramen ovale – both ECHOs were performed on the same day of assessment; VA – verbal autopsy;

Confidential: For Review Only

Table S3: Sensitivity and specificity of the pulse oximeter in detecting CCHD or neonatal sepsis

	**For CCHD detection (n=1317)**	**For detection of neonatal sepsis (n=1317)**
True positives (n)	0	9
True negatives (n)	1289	1259
False positives (n)	26	17
False negatives (n)	2	32
Sensitivity (95% CI) (%)	0 (0-84.2)	21.9 (10.6-37.6)
Specificity (95% CI) (%)	98 (97.1-98.7)	98.6 (97.9-99.2)

The two cases of false negative CCHD were Tetralogy of Fallot and isolated valvar pulmonary stenosis. In both these children, the pulse oximeter readings were above 95% were hand and foot screening.

Confidential: For Review Only

Table S4: Performance metrics of the different machine learning models developed on the clinical, WHO signs and pulse oximetry data

	POx		WHO		POx | WHO		CliML		SeqML		AllML	
	**Train**	**Test**	**Train**	**Test**	**Train**	**Test**	**Train**	**Test**	**Train**	**Test**	**Train**	**Test**
**TP**	7	5	27	17	29	19	37	12	40	23	37	17
**FP**	10	4	5	5	14	9	153	118	165	124	21	15
**FN**	34	22	14	10	12	8	4	15	1	4	4	10
**TN**	739	496	744	495	735	491	596	382	584	376	728	485
**SE**	17	19	66	63	71	70	90	44	98	85	90	63
**SP**	99	99	99	99	98	98	80	76	78	75	97	97
**AUC**	0.58	0.59	0.83	0.81	0.84	0.84	0.85	0.60	0.88	0.80	0.94	0.80
**F1**	0.24	0.28	0.74	0.69	0.69	0.69	0.32	0.15	0.33	0.26	0.75	0.58
**PR**	0.41	0.56	0.84	0.77	0.67	0.68	0.19	0.09	0.20	0.16	0.64	0.53

POx: pulse-oximetry alone; WHO: WHO signs alone; POx | WHO: POx or WHO signs; CliML: ML model for clinical data only; SeqML: sequential use of POx, WHO and CliML; AllML: ML model for the clinical data enriched with WHI signs outcome and POx values (saturations of hand and toe and heart rate); Train: Results for the training set; Test: results for the test set; TP: True Positives; FP: False Positives; FN: False Negatives; TN: True Negatives; SE: Sensitivity; SP: Specificity; AUC: Area under the Curve; F1: F1 score; PR: Precision)

Confidential: For Review Only

Figure S2: SHapley Additive exPlanations (SHAP) analysis for the ML model based on the clinical variables, showing the main feature used in the ML model that contribute most to the decision. The higher the SHAP value, the more important the feature. A positive value contributes to a positive decision (at-risk neonate), while a more negative value suggest the opposite. A red color indicates a high value for the feature, while a blue color corresponds to a low value. For example, from the top features we can see that a low weight and a low gestational age is associated to a more at-risk neonate.

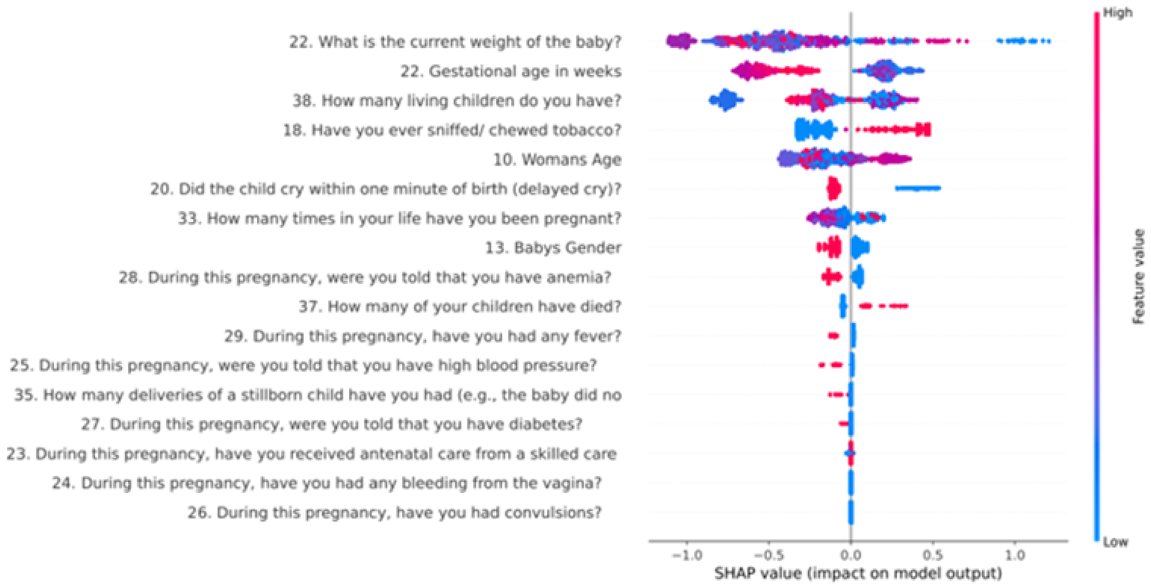

Confidential

Figure S3: ROC analysis for the ML based on clinical variables as well as the sequential use of WHOS, POx and clinical ML

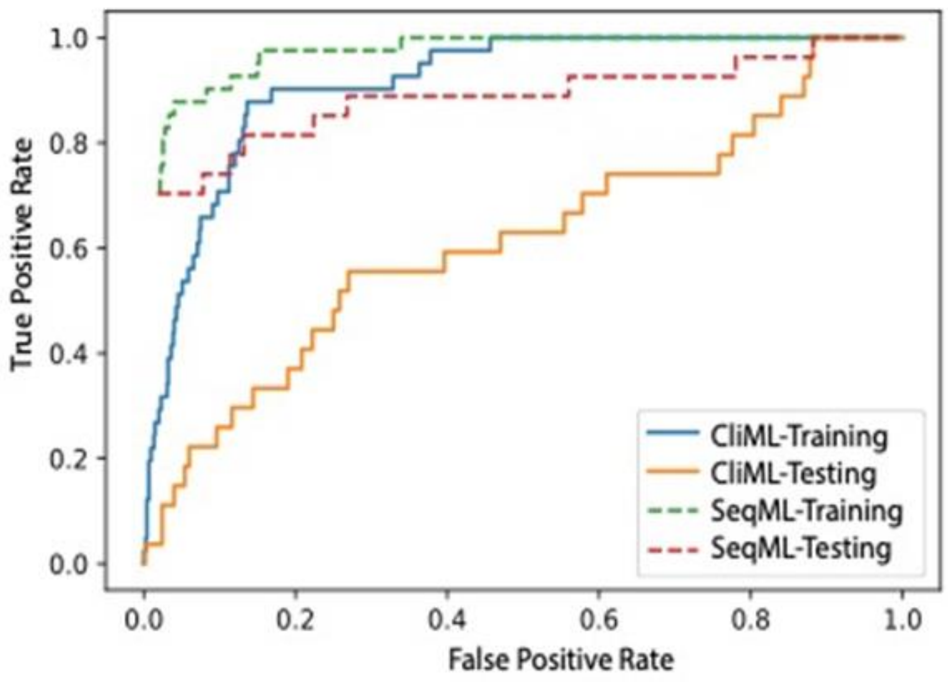

BMJ Paediatrics Open

Can machine learning methods be used for identification of at risk neonates in low resource settings? a prospective cohort study

Journal:	*BMJ Paediatrics Open*
Manuscript ID	bmjpo-2023-002134.R2
Article Type:	Original research
Date Submitted by the Author:	03-Oct-2023
Complete List of Authors:	Hasan, Babar; Sindh Institute of Urology and Transplantation, Department of Pediatrics and Child Health<br>Hoodbhoy, Zahra; Aga Khan University, Department of Pediatrics and Child Health<br>Khan, Amna; The Aga Khan University, Department of Pediatrics and Child Health<br>Nogueira, Mariana; IDIBAPS<br>Bijnens, Bart; IDIBAPS; ICREA<br>Chowdhury, Devyani; The Children's Hospital of Philadelphia
Keywords:	Screening, Neonatology

SCHOLARONE™
Manuscripts

I, the Submitting Author has the right to grant and does grant on behalf of all authors of the Work (as defined in the below author licence), an exclusive licence and/or a non-exclusive licence for contributions from authors who are: i) UK Crown employees; ii) where BMJ has agreed a CC-BY licence shall apply, and/or iii) in accordance with the terms applicable for US Federal Government officers or employees acting as part of their official duties; on a worldwide, perpetual, irrevocable, royalty-free basis to BMJ Publishing Group Ltd ("BMJ") its licensees and where the relevant Journal is co-owned by BMJ to the co-owners of the Journal, to publish the Work in this journal and any other BMJ products and to exploit all rights, as set out in our licence.

The Submitting Author accepts and understands that any supply made under these terms is made by BMJ to the Submitting Author unless you are acting as an employee on behalf of your employer or a postgraduate student of an affiliated institution which is paying any applicable article publishing charge ("APC") for Open Access articles. Where the Submitting Author wishes to make the Work available on an Open Access basis (and intends to pay the relevant APC), the terms of reuse of such Open Access shall be governed by a Creative Commons licence – details of these licences and which Creative Commons licence will apply to this Work are set out in our licence referred to above.

Other than as permitted in any relevant BMJ Author's Self Archiving Policies, I confirm this Work has not been accepted for publication elsewhere, is not being considered for publication elsewhere and does not duplicate material already published. I confirm all authors consent to publication of this Work and authorise the granting of this licence.

Confidential: For Review Only

Title: Can machine learning methods be used for identification of at risk neonates in low resource settings? a prospective cohort study

Authors:

Babar S. Hasan*[1] MBBS, Zahra Hoodbhoy*[2] PhD, Amna Khan[2] BA, Mariana Nogueira[3] PhD, Bart Bijnens PhD[3,4], Devyani Chowdhury[5,6] MD

*Shared first authorship

#Shared senior authorship

[1]Department of Pediatrics and Child Health, Stadium Road, The Aga Khan University, Karachi, Pakistan relocated to Sindh Institute of Urology and Transplantation (SIUT), Karachi, Pakistan

[2]Department of Pediatrics and Child Health, Stadium Road, The Aga Khan University, Karachi, Pakistan

[3]IDIBAPS (August Pi i Sunyer Biomedical Research Institute), Barcelona, Spain

[4]ICREA, Barcelona, Spain

[5]Cardiology Care for Children, Pennsylvania, USA,

[6]Nemours Cardiac Center, Wilmington, DE

Corresponding author:

Babar Hasan
Professor and Chair
Division of Cardiothoracic Sciences
Sindh Institute of Urology and Transplantation (SIUT)
Karachi, Pakistan
Tel No: +92-3362559473
Email address: drbabarhasan@yahoo.com

Confidential: For Review Only

Abstract:

Introduction: Timely identification of at-risk neonates (ARN) in the community is essential to reduce mortality in low resource settings. Tools such as AAP pulse-oximetry (POx) and WHO Young Infants Clinical Signs (WHOS) have high specificity but low sensitivity to identify at risk neonates (ARN). Our aim was assessing the value of POx and WHOS independently, in combination, and with machine learning (ML) from clinical features, to detect ARN in a low- to middle-income country (LMIC)

Methods: This prospective cohort study was conducted in a peri-urban community in Pakistan. Eligible live births were screened using WHOS and POx along with clinical information regarding pregnancy and delivery. The enrolled neonates were followed for 4 weeks of life to assess the vital status. The predictive value to identify ARN, of POx, WHOS and an ML model using maternal and neonatal clinical features was assessed.

Results: Of 1336 neonates, 68 (5%) had adverse outcomes, i.e. sepsis (n=40, 59%), critical congenital heart disease (n=2, 3%), severe persistent pulmonary hypertension (n=1), hospitalization (n=8, 12%) and death (n=17, 25%) assessed at 4 weeks of life. Specificity of POx and WHOS to independently identify ARN was 99%, with sensitivity of 19% and 63% respectively. Combining both improved SE to 70%, keeping SP at 98%. An ML model using clinical variables had 44% SE and 76% SP. A staged assessment, where WHOs, POx and ML are sequentially used for triage increased SE to 85%, keeping SP 75%. Using ML (when WHO and POx negative) for community follow-up detected the majority of ARN.

Conclusion: Classical screening combined with ML, can help maximizing identifying ARN and could be embedded in low-resource clinical settings, thereby improving outcome. Sequential use

Confidential: For Review Only

of classical assessment and clinical ML identifies the most at-risk neonates in the community, still optimizing follow-up clinical care.

Keywords: neonatal wellness; screening; community health workers; machine learning

Confidential: For Review Only

What is already known on this topic?

Two thirds of the global neonatal deaths occur in low middle income countries but tools such as WHO young infants clinical study tool or pulse oximeter have poor sensitivity to identify sick newborns

What this study adds

This study proposes a novel approach that combines highly specific tests like pulse oximeter and WHO with a sensitive machine learning model which uses clinical features in a sequential manner to identify at risk neonates in the community

How this study might affect research, practice or policy

This approach may help front-line health workers in identifying at-risk neonates and by using the strengths of each test, be able to triage and manage these neonates more effectively in low resource settings.

Confidential: For Review Only

Introduction:

With a neonatal mortality rate (NMR) of 42 per 1000 live births, Pakistan accounts for 7% of all global neonatal deaths and ranks 3rd on the list among countries with highest NMR.[1] The top four causes of neonatal deaths are pre-maturity, birth asphyxia, sepsis and congenital anomalies, most of which are congenital heart diseases (CHD).[2]

Newborn screening at few hours of life, for identification of at-risk newborns (ARN), has been proposed as an effective tool to reduce NMR.[3] In high-income countries (HICs), a combination of antenatal ultrasound and newborn screening enables early detection, diagnosis, and intervention to prevent death or disability. Newborn screening, using a few drops of blood for genetic, metabolic and endocrine disorders, and American Academy of Pediatrics (AAP) pulse oximetry (POx) for critical congenital heart defects (CCHDs) are widely implemented in HICs.[4]

A recent systematic review by Plana et al reported that the overall sensitivity of pulse oximetry for the detection of CCHD was 76.3% (95% CI 69.5 to 82.0) (low certainty of the evidence) while the specificity was 99.9% (95% CI 99.7 to 99.9) (high certainty of the evidence).[5] While POx is a highly specific tool to detect CCHD, it will detect other non-cardiac conditions associated with hypoxia, such as neonatal sepsis, hypothermia and persistent pulmonary hypertension,[6, 7] where it has demonstrated a high specificity (>99.9%) but low sensitivity (42%).[8] POx thus has shown a broader utility as a newborn wellness screen, with a good feasibility and acceptability for screening newborns in primary care centers in low middle income countries (LMICs).[9]

A complementary newborn wellness screening tool widely used in LMICs, is the WHO Young Infants Clinical Signs Study Group (WHOS), which identifies seven signs including fever ≥ 38°C, hypothermia (temperature < 35°C), convulsions, lethargy, poor feeding, chest indrawing

Confidential: For Review Only

and tachypnea (respiratory rate > 60 breaths per minute) and has a sensitivity of 74% and specificity of 79% to predict severe illness.[10] Though WHOS and POx have shown only modest sensitivity, these are simple and highly specific screening tools which may be used to identify newborn conditions contributing to NMR in low resource settings.

Given their moderate sensitivity, there remains a need to optimize the performance of these tools and to develop improved strategies by including the use of a wider set of clinical parameters for community-based triage of ARN.[10] The aim of this study was to assess the value of POx and WHOS, used on their own, in combination, and enriched with machine learning (ML) from clinical features, to develop a screening tool for community health workers (CHWs) to identify ARN.

Confidential: For Review Only

Methods:

Study design and participants:

This was an ethics committee approved (Aga Khan University ERC number: 2019-0551-4976) single group prospective cohort study conducted at Ibrahim Hyderi (IH), a peri-urban settlement of approximately 100,000 individuals on the south-east of Karachi, Pakistan, from October 1, 2020 to March 31, 2022. The main occupation of individuals in this area is fishing with 40% below the poverty line.[11] The NMR at this site ranges between 40-45 per 1000 live births.[11] The eligibility criterion was a single live birth where the mother planned to stay in the study catchment area for at least four weeks after delivery.

Sample size estimation:

The sample size was estimated based on the primary outcomes of this study. Neonatal sepsis has been reported to be as high as 14%[2] while the prevalence of CHD in Pakistan is stated to be around 0.9%.[12] With a 95% confidence interval, power of 80% and absolute precision of 0.6%, a frequency of 0.9% generated the maximum sample size of 951 neonates. To account for 5% loss to follow up, the sample size was inflated to 1000 neonates.

Procedures:

The Department of Pediatrics and Child Health at The Aga Khan University (AKU) maintains a demographic and health surveillance system at this site whereby all households are enumerated. Leveraging the surveillance data for identification of pregnant women, we used a nonprobability convenience sampling approach where trained CHWs visited these households to screen eligible women and obtained written informed consent for the participation of their newborn in the research study. All live births registered within 72 hours, where clinical information about

Confidential: For Review Only

the pregnancy and delivery were collected (refer to table S1) while danger signs were assessed using WHOS[10] along with POx (using the Masimo Rad-8 pulse oximeter).

The oxygen saturation (SpO2) was measured on the right hand and foot (30 seconds each) once the device's signal quality index showed stable green bars for >10 seconds. The SpO2 readings were recorded and categorized into green, yellow and red zone as per AAP recommendations (refer to figure 1 and Figure S1).[13] Neonates who had a positive WHOS or fell in the yellow or red zone for POx, were referred for further evaluation and echocardiography with follow up by a physician at the primary health care center (PHC). As per PHC protocol, all clinically sick neonates who were prescribed antibiotics were followed daily for the next 7 days. All neonates were also followed up at four weeks of life to assess the vital status of the child, and hospitalizations, if any, during this period. For all neonatal deaths, a verbal autopsy was performed using the WHO 2016 verbal autopsy instrument by trained research staff[14] and coded by a physician to ascertain cause of death.

Outcomes:

The primary outcome of this study was the identification of ARN, defined as:

- Early neonatal sepsis – Confirmed by a physician after referral based on WHOS or other clinical indications.[10]
- Critical CHD (CCHDs) – Confirmed by echocardiography (transposition of the great arteries, double outlet right ventricle, complex single ventricle physiology, Tetralogy of Fallot, truncus arterious.[15]
- Other cardiac conditions, such as severe persistent pulmonary hypertension (PPHN) (bi-directional patent ductus arteriosus (PDA) and right ventricular dysfunction.)
- Hospitalization/death within the first four weeks of life.

Confidential: For Review Only

Neonates with isolated non-hemodynamically significant CHDs, like small ventricular septal defects (VSD) and atrial septal defects (ASD),[16] were not considered as ARN.

Statistical analysis and Machine Learning:

Conventional data analysis was performed using STATA 16.4 bit. Data was reported as percentages and means +/- standard deviation where appropriate.

For the ML, we used the XGBoost implementation of gradient boosted decision trees.[17] For the first model (AllML), an extensive set of clinical variables (table S1), the measurements obtained from POx, and the outcome of WHOS (pass/fail) was used; for the second model (CliML), a smaller set of clinical variables selected based on a combination of prior expert knowledge on relevant features and the results of a model explainer, ranking variables according to importance for outcome prediction in AllML. We also used the SHapley Additive exPlanations (SHAP)[18] approach to understand the outputs of the ML models. The models were trained on 60% of the data and using 3-fold cross validation while 40% were kept as test set. During the training stage, a grid search was performed to tune model hyperparameters, and those yielding the best cross-validation F1-score were selected. The F1-score and other performance metrics (sensitivity, specificity, precision, the Receiver Operating Characteristic (ROC) and respective Area Under the Curve (AUC) were then computed for the full training and the testing set.

Finally, a sequential use (SeqML) of WHOS and if negative followed by POx and if that too is negative then CliML was assessed. A ROC curve was constructed using a moving threshold on the probability provided by CliML and adding this to the (fixed) sensitivities and specificities of POx after WHOS.

Research ethics statement:

Confidential: For Review Only

This study was approved by The Aga Khan University Ethics Review Committee ERC number: 2019-0551-4976.

Results:

During the study period, 1336 neonates were enrolled from the community (figure 2). Maternal and neonatal characteristics of the study population are described in Table 1. The mean maternal age was 27+/- 5.1 years, 46% (n=618) did not have any formal education, 92%(n=933) had received at least one antenatal visit during the current pregnancy and, one third (n=397) delivered at home.

Table 1: Baseline characteristics of mothers and neonates enrolled in the study

**Maternal characteristics (N=1336)**	
Maternal age (in years), mean ± SD	27+/-5.1
Literacy rate, *n (%)*	618 (46)
Unemployed, *n (%)*	1275 (95)
Number of pregnancies, Median IQR	3 (1-14)
Number of alive children, Median IQR	2 (0-10)
Received antenatal care in the current pregnancy, *n (%)*	1248 (93)
**Neonatal characteristics (N=1336)**	
GA at delivery (in weeks), mean ± SD	39+/-4.5
Preterm	313 (23)
Term	1023 (77)
Place of birth, *n (%)*	
Facility	939 (70)
Home	397 (30)
Mode of delivery, *n (%)*	
Spontaneous vaginal delivery	1015 (76)
Assisted vaginal delivery	3 (<1)
Cesarean section	318 (24)

Confidential: For Review Only

Gender, *n (%)*<br>Female<br>Male	 646 (48)<br>690 (52)
Hours at time of enrollment, Median IQR<br>< 24 hours<br>24-48 hours<br>48 -72 hours<br>>72 hours	48.2 (0-141)<br>256 (19)<br>462 (34.5)<br>389 (29.1)<br>229 (17.1)
Weight at enrollment in kg, Median IQR	2.84 (1.19-5.72)
Difference in hours between pulse oximeter screening and echocardiography<br>< 24 hours<br>24-72 hours<br>>72 hours	 <br>42 (60)<br>14 (20)<br>14 (20)

For the final outcome at four weeks follow up, neonates were labeled as *well* or *ARN*. There were 19 neonates who were excluded from the analysis due to refusal for follow up. We identified 68/1317 (5.1%) ARN. These included 40 cases (59%) of neonatal sepsis, 2 cases (3%) of CCHD (both Tetralogies of Fallot, one also with sepsis), 1 case (1.5%) of severe PPHN (bi-directional PDA with right ventricular dysfunction), 8 (11.5%) hospitalizations and 17 deaths (25%). The detailed characteristics of the study participants who died are described in Table S2. Of the neonatal deaths (n=17), 76% (n=13) passed both WHOS and pulse oximeter screening. Five of these (30%) had the screening performed at less than 24 hours of life. Five were early neonatal deaths with approximately 2.5 days between enrollment and death while for the 12 late neonatal deaths, the median difference between enrollment and death was 16.5 days (IQR 6-26.5).

The distribution of the neonates according to WHOS and POx screening is shown in figure 3 with more details in table S3.

ARN screening performance

Confidential: For Review Only

1317 neonates were included in the predictive modeling to assess ARN. The training set (60%) consisted of 790 neonates while the test set included 527. Table S4 summarizes all results. As expected, WHOS (absent/present) and POx (pass/fail) showed a high specificity, but only moderate sensitivity to detect ARN. Combining both improved overall sensitivity as compared to the singular tests, while preserving specificity. When comparing the results in training and test set, they showed similar performance, confirming their stable performance in this population.

The ML model based on the clinical variables (CliML) showed good sensitivity/specificity on training, but much worse performance during testing, indicating overfitting.

AllML, trained on all assessed information at once, showed excellent performance at training, but reduced sensitivity in the test set indicating overfitting,. The variables that contributed most to the decision included maternal features such as tobacco use, previous pregnancies, child deaths and pregnancy complication, as well as neonatal features, such as gender, gestational age at delivery and birthweight as indicated by the SHAP analysis (figure S2).

To find a balance between the high specificity of WHOS and POx and the potentially higher sensitivity of CliML, a sequential testing approach, composed of first assessing WHOS, followed by POx and CliML to further identify ARN was investigated (figure 4). Despite some overfitting during training, SeqML clearly showed increased sensitivity in the test set, as compared to all other approaches. Figure S3 shows the ROC curve for both CliML as well as the sequential approach of SeqML.

Confidential: For Review Only

Discussion:

High NMR in LMIC is an important public health problem that can be addressed by timely detection of ARN. It is important to identify a community based implementation framework where neonates can be appropriately followed by confirmatory testing and affordable management pathways.[19]

This study showed that a sequentially deployed strategy for identification of ARN, can outperform the traditional approach of using only POx or WHO signs. The model identifies a maximal number of ARN and can be implemented in a staged manner where those failing WHOS and POx can be referred sooner to a higher-level care facility, while intermediate risk neonates detected by the SeqML are monitored at home by a CHW.

WHOS has modest sensitivity to detect non-infectious etiologies. In the framework presented by the Global Initiative for Children's cardiac Surgery, PO and WHOS are a part of basic infrastructure for timely outpatient screening for cardiac disease.[20] POx is recommended for screening for CCHD and is also able to detect other hypoxia causing non-cardiac conditions.[13, 21] This has led to proposed recommendations of the WHO Integrated Management of Childhood Illnesses (IMCI) guidelines for detecting severe pneumonia to include oxygen saturation of <93% as a point for referral.[22] In this study, POx is highly specific for identifying ARN, but has a poor sensitivity when used stand-alone. Amongst factors that might contribute to this is the timing of the screening as while the ductus arteriosus is still open there is a higher sensitivity to detect life-threatening CCHD.[23, 24] In the current study, there were four cases who were screened > 24 hours after birth, did not have WHOS and passed the POx screening, but died within 7-14 days of birth at home where cause of death could not be ascertained. It may be possible that an underlying cardiac condition, especially left sided lesions, may have been

Confidential: For Review Only

missed due to late neonatal screening. Additionally, the POx screening devices used only provided readings of the saturation values and heart rate and did not have visual feedback on acquired waveforms. While out of the scope of this manuscript, when examining the stored waveforms, it was observed that the quality of tracing might have also contributed to the lower sensitivity.

Besides the outcome of WHOS and POx for screening, the study showed that their combination allows to increase sensitivity while maintaining high specificity, indicating detecting other etiologies associated with ARN. However, the modest sensitivity of the combination of both indicates that not all ARN are identified. CliML provided predictive information, but only at modest sensitivity and specificity. Training a singular ML model (AllML) using all available information at once showed potential but had problems with generalizability given that the performance in the test data was inferior to the training. The sequential screening model (SeqML) where WHOS, a specific test first identifies neonates at risk of sepsis and then identifies etiologies associated with decreased saturations, through POx, and finally identifies those at higher risks using an ML algorithm based on clinical information, was most successful in identifying ARN. This approach optimizes a staged follow-up given that WHOS and POx identify those that clearly need urgent medical attention, while the clinical data that can be embedded and entered in an application on the CHW's mobile phone that identifies an increased risk without unique etiology, but that could benefit from closer follow-up. The drawback of this sequential approach is the high number of false positives (23.6%, n=312). Rather than immediate referral of these cases, we propose that children who pass WHOS and POx, but fail CliML, should have regular follow ups by the CHW as per the WHO guidelines for postnatal care and refer if needed.(days 3, 7, 14 and 42 days of life).[25] During these follow ups, the CHW can use WHOS and POx as tools to reassess the neonate and refer to a care facility if deterioration occurs. As seen in the simplified

Confidential: For Review Only

antibiotic therapy trial, home based follow up by CHWs at assigned days during the first 2 week of life, helped with timely identification of sick neonates.[26] Further, the mother can also be counseled regarding danger signs in the neonate and the need to seek help immediately.[27] These approaches may be an effective strategy for task sharing and optimizing resource utilization s without overburdening the health system with unnecessary referrals.[28] This proposed data-driven and staged approach increases the sensitivity, while keeping the false positives manageable in the healthcare setting.

There are some limitations of the current study. The proposed clinical ML model needs to be externally validated in a larger population to assess its performance, given the difference between train and test performance. Additionally, the AllML model should be further explored and trained with larger datasets to assess its value as compared to the proposed sequential approach. The complementary value of using the actual values of hand and foot oxygen saturation together with heart rate, instead of the pass/fail information from the POx may provide important information. After enrollment in the study, 73/1955 children died before any screening was performed, which may indicate a selection bias and may contribute towards the relatively low NMR (12.7/1000 livebirths) seen in this study. Also, the current model includes clinical parameters such as gestational age, which was calculated based on the self-reported date of last menstrual period which is known to have low reliability. Furthermore, POx was performed using one particular device only. Future research would require external validation of the sequential decision-making approach using the ML model in a community-based setting in improving detection of ARN.

Conclusion:

Confidential: For Review Only

In this study, the value of different screening strategies to detect ARN was assessed. While assessing WHOS and performing POx are highly specific, adding an ML model based on clinical (maternal, neonatal and pregnancy-related) parameters could increase sensitivity. The increase in false-positives would be easily managed if a staged, task sharing approach involving community-based triage, close follow-up and early referral if needed by CHWs, is adopted. This model is also in alignment with the framework presented to decrease the global burden of childhood mortality due to non-communicable diseases.

Confidential: For Review Only

Figure legends:

Figure 1: Screening algorithm used by community health workers

Figure 2: Enrollment of study participants

Figure 3: Distribution of neonates who failed the WHO signs and pulse oximeter screening

Figure 4: Potential integration of the machine learning model in community-based workflows

Acknowledgements:

We would like to thank the study staff and the study participants for their contribution in this work. We would also like to acknowledge our funders including Islamic Development Bank, Bill and Melinda Gates Foundation and the Fundació La Marató de TV.

Data sharing:

Individual participant de-identified data will be made available upon request to the study authors 12 months after publication of this manuscript**.**

Patient and public involvement: Patients and/or the public were not involved in the design, or conduct, or reporting, or dissemination plans of this research.

Funding support: This work has been funded by the Islamic Development Bank (10/HS/ 650 /441), the Bill and Melinda Gates Foundation (INV-021528), and the Fundació La Marató de TV3 (Ref 202016-30-31).

Contributors: BH, ZH and DC designed and planned the study. BH, ZH, AK and DC implemented the study and supervised data collection. BB, MN, AK and ZH conducted the analyses. BH, ZH, AK, MN, BB and DC wrote and edited the manuscript. BH as the guarantor of the overall content.

Competing interests: None declared

Confidential: For Review Only

References:

1. NIPS I. Pakistan demographic and health survey 2017-18. Islamabad, Pakistan, and Rockville, Maryland, USA: NIPS, ICF. 2019.
2. Hug L, Alexander M, You D, Alkema L, for Child UI-aG. National, regional, and global levels and trends in neonatal mortality between 1990 and 2017, with scenario-based projections to 2030: a systematic analysis. The Lancet Global Health. 2019;7(6):e710-e20.
3. Therrell BL, Padilla CD, Loeber JG, Kneisser I, Saadallah A, Borrajo GJ, et al., editors. Current status of newborn screening worldwide: 2015. Seminars in perinatology; 2015: Elsevier.
4. Watson MS, Lloyd-Puryear MA, Howell RR. The Progress and Future of US Newborn Screening. International Journal of Neonatal Screening. 2022;8(3):41.
5. Plana MN, Zamora J, Suresh G, Fernandez-Pineda L, Thangaratinam S, Ewer AK. Pulse oximetry screening for critical congenital heart defects. Cochrane Database of Systematic Reviews. 2018(3).
6. Oster ME, Aucott SW, Glidewell J, Hackell J, Kochilas L, Martin GR, et al. Lessons learned from newborn screening for critical congenital heart defects. Pediatrics. 2016;137(5).
7. Williams KB, Horst M, Hollinger EA, Freedman J, Demczko MM, Chowdhury D. Newborn Pulse Oximetry for Infants Born Out-of-Hospital. Pediatrics. 2021;148(4).
8. Jawin V, Ang H-L, Omar A, Thong M-K. Beyond critical congenital heart disease: newborn screening using pulse oximetry for neonatal sepsis and respiratory diseases in a middle-income country. PloS one. 2015;10(9):e0137580.
9. Emdin CA, Mir F, Sultana S, Kazi A, Zaidi AK, Dimitris MC, et al. Utility and feasibility of integrating pulse oximetry into the routine assessment of young infants at primary care clinics in Karachi, Pakistan: a cross-sectional study. BMC pediatrics. 2015;15(1):1-11.
10. Group YICSS. Clinical signs that predict severe illness in children under age 2 months: a multicentre study. The Lancet. 2008;371(9607):135-42.
11. Ilyas M, Naeem K, Fatima U, Nisar MI, Kazi AM, Jehan F, et al. Profile: health and demographic surveillance system in peri-urban areas of Karachi, Pakistan. Gates Open Research. 2018;2(2):2.
12. Agadoorappa P, Oddie S, Pawson N, Sheridon E. Do pakistani babies have more congenital heart defects? Preliminary findings from our birth cohort study. Archives of Disease in Childhood. 2011;96(Suppl 1):A35-A.
13. Martin GR, Ewer AK, Gaviglio A, Hom LA, Saarinen A, Sontag M, et al. Updated strategies for pulse oximetry screening for critical congenital heart disease. Pediatrics. 2020;146(1).
14. Nichols EK, Byass P, Chandramohan D, Clark SJ, Flaxman AD, Jakob R, et al. The WHO 2016 verbal autopsy instrument: an international standard suitable for automated analysis by InterVA, InSilicoVA, and Tariff 2.0. PLoS medicine. 2018;15(1):e1002486.
15. CDC. Critical Congenital Heart Defects 2022 [cited 2022 May 6]. Available from: https://www.cdc.gov/ncbddd/heartdefects/cchd-facts.html.
16. Prabhakaran D, Anand S, Watkins D, Gaziano T, Wu Y, Mbanya JC, et al. Cardiovascular, respiratory, and related disorders: key messages from Disease Control Priorities. The Lancet. 2018;391(10126):1224-36.
17. Chen T, Guestrin C, editors. Xgboost: A scalable tree boosting system. Proceedings of the 22nd acm sigkdd international conference on knowledge discovery and data mining; 2016.

Confidential: For Review Only

18. Lundberg SM, Lee S-I. A unified approach to interpreting model predictions. Advances in neural information processing systems. 2017;30.
19. Padilla CD, Krotoski D, Therrell Jr BL, editors. Newborn screening progress in developing countries—overcoming internal barriers. Seminars in perinatology; 2010: Elsevier.
20. Hasan BS, Bhatti A, Mohsin S, Barach P, Ahmed E, Ali S, et al. Recommendations for developing effective and safe paediatric and congenital heart disease services in low-income and middle-income countries: a public health framework. BMJ Global Health. 2023;8(5):e012049.
21. Ewer AK. Pulse oximetry screening for critical congenital heart defects in newborn infants: should it be routine? Archives of Disease in Childhood-Fetal and Neonatal Edition. 2014;99(1):F93-F5.
22. McCollum ED, Ginsburg AS. Outpatient management of children with World Health Organization chest indrawing pneumonia: implementation risks and proposed solutions. Clinical Infectious Diseases. 2017;65(9):1560-4.
23. Ng B, Hokanson J. Missed congenital heart disease in neonates. Congenital heart disease. 2010;5(3):292-6.
24. Liske MR, Greeley CS, Law DJ, Reich JD, Morrow WR, Baldwin HS, et al. Report of the Tennessee task force on screening newborn infants for critical congenital heart disease. Pediatrics. 2006;118(4):e1250-e6.
25. Organization WH. WHO recommendations on postnatal care of the mother and newborn: World Health Organization; 2014.
26. Mir F, Nisar I, Tikmani SS, Baloch B, Shakoor S, Jehan F, et al. Simplified antibiotic regimens for treatment of clinical severe infection in the outpatient setting when referral is not possible for young infants in Pakistan (Simplified Antibiotic Therapy Trial [SATT]): a randomised, open-label, equivalence trial. The Lancet Global Health. 2017;5(2):e177-e85.
27. Rahman AE, Herrera S, Rubayet S, Banik G, Hasan R, Ahsan Z, et al. Managing possible serious bacterial infection of young infants where referral is not possible: Lessons from the early implementation experience in Kushtia District learning laboratory, Bangladesh. PloS one. 2020;15(5):e0232675.

Confidential: For Review Only

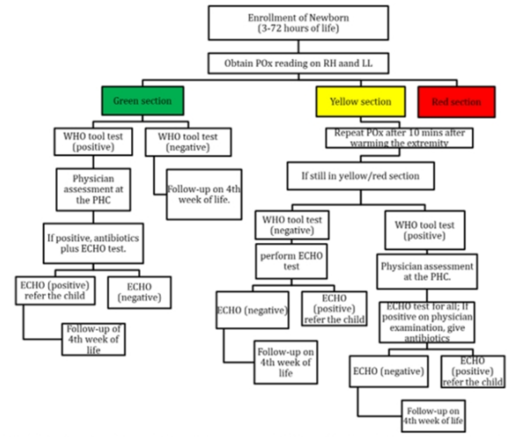

*RH – right hand, LL – lower limb, Pox – pulse oximeter screening, WHO – WHO signs, ECHO – echocardiography, PHC– primary health center. Any child who failed pulse oximeter screening and/or WHO signs by the community health worker at the home assessment was recommended an ECHO

108x60mm (300 x 300 DPI)

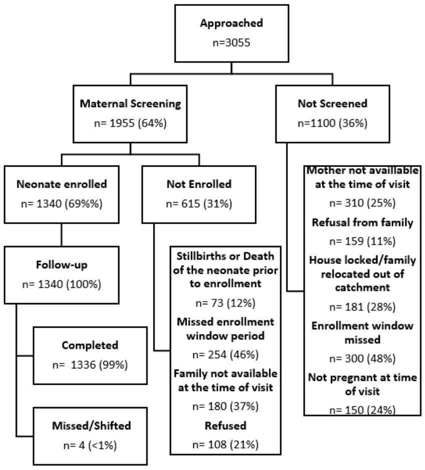

108x60mm (300 x 300 DPI)

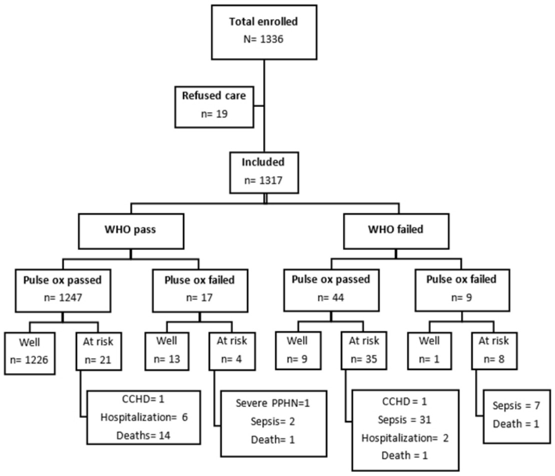

108x60mm (300 x 300 DPI)

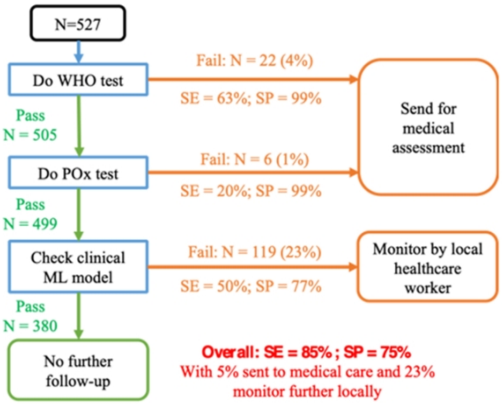

108x60mm (300 x 300 DPI)

SUPPLEMENTARY MATERIAL

Figure S1: CHD screening table using pulse oximetry value on right hand and either foot

Right Hand	Either Foot											
100	100	99	98	97	96	95	94	93	92	91	90	<90
99	100	99	98	97	96	95	94	93	92	91	90	<90
98	100	99	98	97	96	95	94	93	92	91	90	<90
97	100	99	98	97	96	95	94	93	92	91	90	<90
96	100	99	98	97	96	95	94	93	92	91	90	<90
95	100	99	98	97	96	95	94	93	92	91	90	<90
94	100	99	98	97	96	95	94	93	92	91	90	<90
93	100	99	98	97	96	95	94	93	92	91	90	<90
92	100	99	98	97	96	95	94	93	92	91	90	<90
91	100	99	98	97	96	95	94	93	92	91	90	<90
90	100	99	98	97	96	95	94	93	92	91	90	<90
<90	100	99	98	97	96	95	94	93	92	91	90	<90

For Review Only

Table S1: For AllML, the following clinical variables were used:

Group	Variable
Maternal characteristics	Age
	Years of education
	Smoking
	Sniffing/chewing of tobacco
	Chewing of betel nut
	Cardiac disease
	Diabetes
	Thyroid disease
	Any type of cancer
	Epilepsy
Obstetric History	Number of pregnancies
	Number of pregnancies ended before 7 months
	Number of stillborn
	Number of deliveries
	Number of live deliveries
	Number of children died during delivery
	Number of living children
	Number of children died
	Number of children with congenital heart disease
	Number of children with any heart surgery
Current pregnancy characteristics	Received antenatal care from a skilled care
	Vaginal bleeding
	High blood pressure
	Convulsions
	Diabetes
	Anemia
	Fever
	Blood transfusion
	Overnight admission to a health facility
Neonatal characteristics	Gestational age
	Delayed cry
	Weight
	Gender
	Place of birth
	Mode of delivery

Confidential: For Review Only

Table S2: Details of the study participants who died at four weeks of age

S.No	Mode of delivery	Place of delivery	GA at time of delivery (weeks)	Birth weight (kg)	Age at time of enrollment (hours)	WHO Signs	Pulse Oxi-meter	ECHO done	Age at death (in days)	Place of death	Cause of death
**1**	SVD	Clinic	32	2.53	43	Pass	Pass	No	8	Hospital	Neonatal sepsis
**2**	SVD	Hospital	40	2.59	36	Pass	Pass	No	19	Home	Neonatal sepsis
**3**	SVD	Home	40	1.92	6	Fail	Fail	Yes*	13	Home	Neonatal sepsis
**4**	SVD	Hospital	25	1.19	49	Pass	Pass	No	16	Home	Pre-term related complicatio n
**5**	SVD	Clinic	38	2.00	11	Fail	Pass	No (refused)	6	Hospital	Neonatal sepsis
**6**	SVD	Hospital	35	1.92	34	Pass	Pass	No	3	Home	Not determined after VA
**7**	SVD	Hospital	40	2.86	69	Fail	Pass	No (refused)	3	Hospital	Neonatal sepsis
**8**	SVD	Clinic	38	2.96	58	Pass	Pass	No	6	Hospital	Neonatal tetanus
**9**	SVD	Hospital	33	2.34	78	Pass	Pass	No	30	Home	Not determined after VA
**10**	SVD	Clinic	40	4.21	21	Pass	Pass	No	5	Home	Not determined after VA
**11**	SVD	Home	34	1.86	37	Pass	Pass	No	9	Home	Not determined after VA
**12**	SVD	Home	39	2.76	11	Pass	Pass	No	11	Home	Neonatal sepsis
**13**	SVD	Home	51	2.08	31	Pass	Pass	No	28	Hospital	Neonatal sepsis

**14**	Cesarean section	Hospital	36	3.29	96	Pass	Pass	No	20	Home	Not determined after VA
**15**	SVD	Home	40	2.73	18	Pass	Pass	No	26	Home	Neonatal sepsis
**16**	SVD	Hospital	39	2.56	67	Pass	Fail	Yes^	21	Home	Neonatal pneumonia
**17**	Cesarean section	Hospital	36	2.56	94	Pass	Pass	No	18	Home	Neonatal sepsis

*Severe tricuspid regurgitation, ^patent foramen ovale – both ECHOs were performed on the same day of assessment; VA – verbal autopsy;

Confidential: For Review Only

Table S3: Sensitivity and specificity of the pulse oximeter in detecting CCHD or neonatal sepsis

	**For CCHD detection (n=1317)**	**For detection of neonatal sepsis (n=1317)**
True positives (n)	0	9
True negatives (n)	1289	1259
False positives (n)	26	17
False negatives (n)	2	32
Sensitivity (95% CI) (%)	0 (0-84.2)	21.9 (10.6-37.6)
Specificity (95% CI) (%)	98 (97.1-98.7)	98.6 (97.9-99.2)

The two cases of false negative CCHD were Tetralogy of Fallot and isolated valvar pulmonary stenosis. In both these children, the pulse oximeter readings were above 95% were hand and foot screening.

Confidential: For Review Only

Table S4: Performance metrics of the different machine learning models developed on the clinical, WHO signs and pulse oximetry data

	POx		WHO		POx | WHO		CliML		SeqML		AllML	
	Train	Test	Train	Test	Train	Test	Train	Test	Train	Test	Train	Test
**TP**	7	5	27	17	29	19	37	12	40	23	37	17
**FP**	10	4	5	5	14	9	153	118	165	124	21	15
**FN**	34	22	14	10	12	8	4	15	1	4	4	10
**TN**	739	496	744	495	735	491	596	382	584	376	728	485
**SE**	17	19	66	63	71	70	90	44	98	85	90	63
**SP**	99	99	99	99	98	98	80	76	78	75	97	97
**AUC**	0.58	0.59	0.83	0.81	0.84	0.84	0.85	0.60	0.88	0.80	0.94	0.80
**F1**	0.24	0.28	0.74	0.69	0.69	0.69	0.32	0.15	0.33	0.26	0.75	0.58
**PR**	0.41	0.56	0.84	0.77	0.67	0.68	0.19	0.09	0.20	0.16	0.64	0.53

POx: pulse-oximetry alone; WHO: WHO signs alone; POx | WHO: POx or WHO signs; CliML: ML model for clinical data only; SeqML: sequential use of POx, WHO and CliML; AllML: ML model for the clinical data enriched with WHI signs outcome and POx values (saturations of hand and toe and heart rate); Train: Results for the training set; Test: results for the test set; TP: True Positives; FP: False Positives; FN: False Negatives; TN: True Negatives; SE: Sensitivity; SP: Specificity; AUC: Area under the Curve; F1: F1 score; PR: Precision)

Confidential: For Review Only

Figure S2: SHapley Additive exPlanations (SHAP) analysis for the ML model based on the clinical variables, showing the main feature used in the ML model that contribute most to the decision. The higher the SHAP value, the more important the feature. A positive value contributes to a positive decision (at-risk neonate), while a more negative value suggest the opposite. A red color indicates a high value for the feature, while a blue color corresponds to a low value. For example, from the top features we can see that a low weight and a low gestational age is associated to a more at-risk neonate.

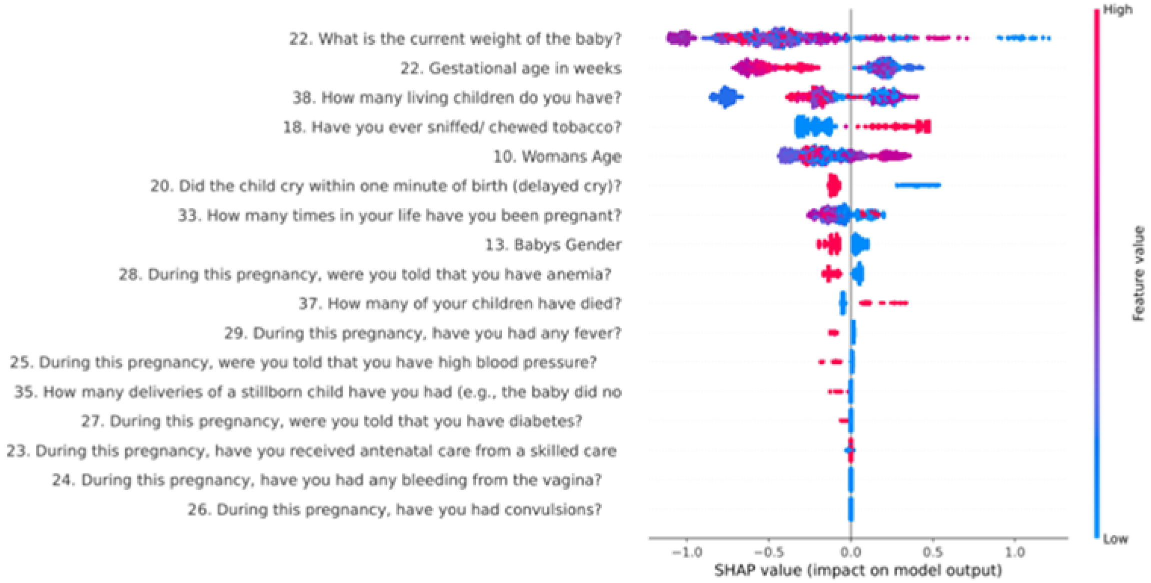

Confidential

Figure S3: ROC analysis for the ML based on clinical variables as well as the sequential use of WHOS, POx and clinical ML

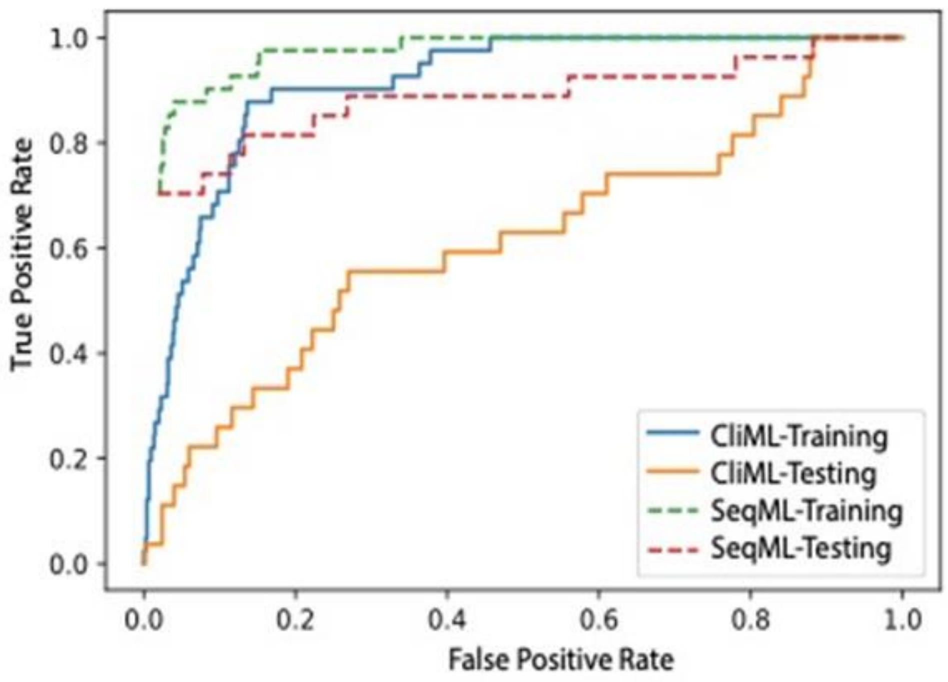

Supplement: Author's manuscript [file bmjpo-2023-002134.draft_revisions.pdf]
